# Are We There Yet? A Roadmap of Network Visualization from Surveys to Task Taxonomies

**DOI:** 10.1111/cgf.14794

**Published:** 2023-04-04

**Authors:** Velitchko Filipov, Alessio Arleo, Silvia Miksch

**Affiliations:** ^1^ TU Wien, CVAST Vienna Austria

**Keywords:** information visualization, network visualization, visual analytics

## Abstract

Networks are abstract and ubiquitous data structures, defined as a set of data points and relationships between them. Network visualization provides meaningful representations of these data, supporting researchers in understanding the connections, gathering insights, and detecting and identifying unexpected patterns. Research in this field is focusing on increasingly challenging problems, such as visualizing dynamic, complex, multivariate, and geospatial networked data. This ever‐growing, and widely varied, body of research led to several surveys being published, each covering one or more disciplines of network visualization. Despite this effort, the variety and complexity of this research represents an obstacle when surveying the domain and building a comprehensive overview of the literature. Furthermore, there exists a lack of clarification and uniformity between the terminology used in each of the surveys, which requires further effort when mapping and categorizing the plethora of different visualization techniques and approaches. In this paper, we aim at providing researchers and practitioners alike with a “roadmap” detailing the current research trends in the field of network visualization. We design our contribution as a meta‐survey where we discuss, summarize, and categorize recent surveys and task taxonomies published in the context of network visualization. We identify more and less saturated disciplines of research and consolidate the terminology used in the surveyed literature. We also survey the available task taxonomies, providing a comprehensive analysis of their varying support to each network visualization discipline and by establishing and discussing a classification for the individual tasks. With this combined analysis of surveys and task taxonomies, we provide an overarching structure of the field, from which we extrapolate the current state of research and promising directions for future work.

## Graphical abstract

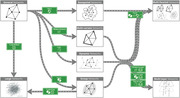

## 1 Introduction

A network is an abstract and ubiquitous data structure, defined, in its simplest form, as the combination of a set of data points and the relationships between them. Due to its simple yet flexible nature, it found its way to a wide range of applications in diverse problem domains. Network visualization is a research field concerned with providing meaningful representations of networked data, supporting researchers in understanding the connections, gathering insights, and detecting and identifying unexpected patterns [BCD*10].

Over the last two decades, the field of network visualization has been building up momentum and developing rapidly, focusing its research on increasingly challenging disciplines, such as the visualization of dynamic, complex, multi‐variate, geospatial network data. The variety and diversity of this research represents an obstacle for researchers and practitioners surveying the domain, and, more specifically, when building a comprehensive overview of the literature in the field. To address this problem, numerous surveys have been published on these different disciplines of network visualization, that is, large network visualization [vLKS*11], dynamic network visualization [BBDW17], multi‐variate network visualization [NMSL19], and others [SSK14, HSS15, VBW17, NMSL19, MGM*19, SYPB21]. While immensely helpful in systematically exploring and categorizing research in their own disciplines, similar approaches and techniques have been classified by multiple surveys in different ways and under diverse names, ultimately resulting in a lack of clarification and uniformity between the terminology used. For example, the concept of *juxtaposition* has been referred to as “small multiples”, “static flipbooks”, or “[visualization of] multiple timeslices” in the context of dynamic network visualization. This requires further effort when mapping and categorizing the plethora of different visualization techniques and approaches. Within this context, our motivation is closely aligned to the one expressed by McNabb et al. [ML17], outlining the need for a “quantum step” in survey literature to provide an overarching structure of the field, explore saturated areas of research, and emphasize directions for future work. Questions such as “What has already been done?” and “What areas in the domain have yet to be explored?” are common occurrences that we aim to address in our work.

In this paper, we present a meta‐survey with the goal of providing a *roadmap* detailing the different research directions in the field of network visualization and the relationships between them. A roadmap is a form of map that provides an overview of the landscape and details navigational routes, points of interest, roads, and boundaries. A roadmap can also be defined as a strategic plan outlining the desired outcome (or goal), the respective challenges, and defining the necessary steps required to reach it. In the same way that a map provides navigational routes, we want to support both newcomers and seasoned explorers of the field with a concise yet accessible view of the research field of network visualization, detailing the most relevant research trends and highlighting more and less conspicuous relationships between different visualization disciplines. Following our roadmap metaphor, we categorize the research in the field based on two major landmarks: *surveys* (or state‐of‐the‐art reports) and *task taxonomies*. We start by reviewing the surveys and the proposed categorizations, examining their differences and commonalities, cross‐cutting challenges and research trends, and discussing the overlaps and inconsistencies in terminology across different publications. Second, we extend our review to task taxonomies. Task taxonomies are concepts that are widely used for comparing and evaluating the efficacy of techniques and approaches [SYPB21]. Several surveys across different disciplines outline the need for a standardized set of tasks, often in response to the lack of a task taxonomy sufficiently expressive for their type of data. We explore the individual task categorizations and outline their design and evaluation process. We present a classification of the proposed tasks and evaluate their support for the different visualization disciplines matching the relationships we identified in our roadmap metaphor. Within this context, our contributions are as follows:

- Provide a structured overview of surveys published in the field of network visualization based on a literature search oriented towards highlighting more and less saturated research directions, current cross‐cutting challenges, and discussing the evolution of the field (see Sections 3 and 4);
- Outline overlaps, similarities, and inconsistencies in terminology between the categorizations provided by different surveys in order to support a common dictionary of the field (see Section 5);
- Explore the landscape of task taxonomies associated with different network visualization disciplines, identify which have a well‐established (generalized or specialized) or missing taxonomy, and classify the individual tasks as topology, analysis, or network facet tasks (see Sections 6 and 7);
- Summarize the open challenges and directions for future research as a basis for a discussion about future perspectives for the field of network visualization (see Section 8).

## 2 A *Roadmap* for Network Visualization

**Figure 1:**
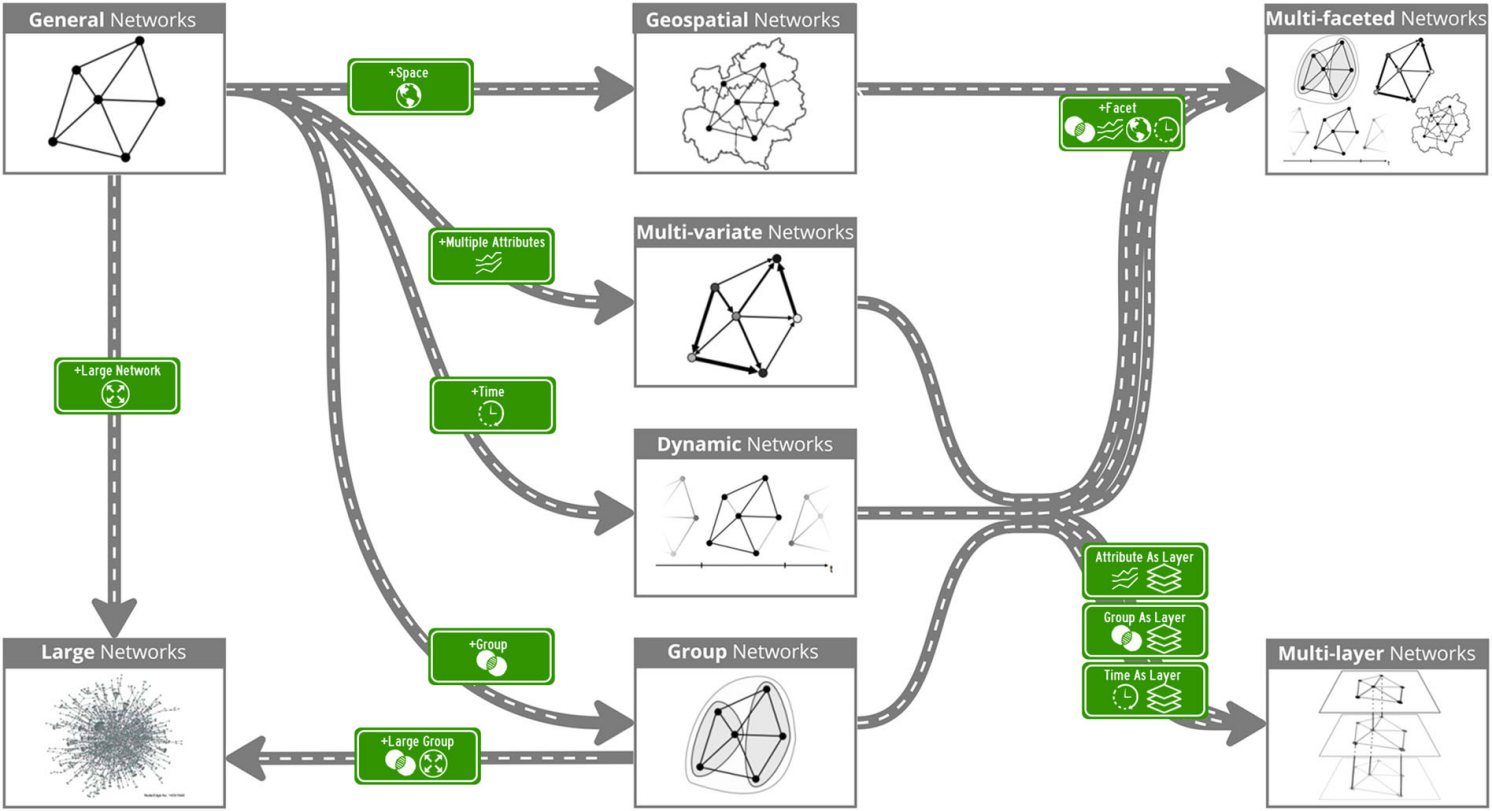
Our roadmap metaphor shows the individual disciplines as nodes with a title and gray border, and their relationships, identified in our literature search, as labelled roads connecting them (Section 2.2). Directed arrows represent inheritance relationships between disciplines. A source discipline can be extended to the target by considering extensions to the data type, depicted by the green road signs.

As network visualization significantly expanded over the last two decades, navigating it might challenge both expert and novice researchers. The difficulty in properly orienting in this field inspired us with the roadmap metaphor (see Figure 1). In the same way a map is generally used to plan a journey, our roadmap is designed to guide and support researchers in finding inspiration, competition, or the most appropriate tasks for their evaluations. In this section, we present our research methodology, discuss our classification, and illustrate our roadmap metaphor for network visualization. We define the terminology we used to categorize publications, elaborate on the scope of our literature review, and discuss related work.

In the context of our work we use the terms **network** and **graph**, **nodes** and **vertices**, and **links** and **edges** interchangeably. While there is a semantic difference between *graph* and *network* (i.e. graphs are mathematical concepts, whereas networks are more general, often referring to real‐world systems), in visualization literature these two terms are used interchangeably and the keywords associated with the publications reflect this. For this purpose, we use *graph* and *network visualization* as keywords when conducting the literature search to retrieve a more extensive set of publications.

### 2.1 Research methodology

Our literature search is an iterative process made up of a sequence of refinement cycles, and we incorporate aspects of the PRISMA statement [PMB*21] in our methodology. The scope of our meta‐survey is strongly oriented to both the visualization and graph drawing communities. We, therefore, reviewed venues more closely related to this audience, including the *IEEE Visualization Conference (VIS)*, *Eurographics Conference on Visualization (EuroVis)*, and *International Symposium on Graph Drawing and Network Visualization (GD)* proceedings, or journals such as *Computer Graphics Forum (CGF), IEEE Transactions of Visualization and Computer Graphics (TVCG), Journal of Graph Drawing and Algorithms (JGAA)*. We then extended our search to include a broader range of venues and journals about general visualization topics, such as *Journal of Visualization (Springer), Advanced Visual Interfaces (AVI), Visual Informatics (Elsevier)*. In Table 1 we report the number of papers included in this meta‐survey with the corresponding venue where it was originally published: we grouped as “Other” all venues which contributed to this survey with a single relevant publication. For the complete set of literature that we gathered in the scope of this work we refer to the supplementary material. We take advantage of the SurVis [BKW15] project to provide readers access to our tagged database of papers (see our online material [PAP] for more information).

**Table 1:** A list of the sources and venues we reviewed along with the corresponding number of papers included in our meta‐survey. Only venues with a paper count greater than zero are shown. Venues and journals in “Other” contribute with only one publication each.

Publication venue	Count
TVCG	9
CGF	9
EuroVis	3
VIS	2
Information visualization	3
AVI	2
Other	15
**Total**	43

Our literature search process started with a small set of surveys that were previously known to the authors. This initial group was extended by querying search engines, such as *Google Scholar* [SCH], *IEEE Explore* [IEX], *ACM Digital Library* [ACM], *ResearchGate* [RES], or *Scopus* [SCO] for publications tagged with the following combinations of keywords:

*(“Survey” | “State‐of‐the‐Art” | “Taxonomy” | “Design Space”)*

*&*

*(“Graph Visualization” | “Network Visualization”)*.

Our aim was to include as many potential publications as possible in this step. The publications gathered in this step were identified as systematic reviews due to their proposed categorization and thorough survey of state‐of‐the‐art approaches. We additionally queried for more specialized surveys and taxonomies adding specific keywords that we identified from the literature we found along our search (e.g. “Multi‐variate”, “Dynamic”, etc.). We verified that all the queried publications fit our inclusion criteria (see Section 2.2). The resulting publications were then investigated in more detail, tagged by the co‐authors, and categorized according to our roadmap metaphor (see Section 2.3).

After this initial step we conducted a forward and reverse lookup of papers citing or cited by our initial set of selected publications in order to discover any new reviews that would fit our topic and to verify that the literature we gathered was extensive and complete. For all the new papers discovered in this second stage, we again checked them against our inclusion criteria and categorized them accordingly. Before checking for inclusion, the combination of these two search steps yield a total of **152** papers. From these, we excluded systematic reviews of graph algorithms (28), books on methods and models of graph drawing (8), studies and evaluations (11), and surveys from other domains not focusing on visualization, such as, cryptography, biology, sociology (11), related work that was retrieved due to the generic keywords (39). Others (12) were excluded as they were out of our time window of interest (2000–2021). Finally, a total of **43** publications met all our inclusion criteria: we categorize them according to Section 2.3 and describe them in greater detail in their respective sections (see Sections 3 and 6).

### 2.2 Meta‐survey scope

In the following, we describe the inclusion criteria for surveys and taxonomies we consider in the scope of our work and present examples of literature that were included or excluded from our research. Among all paper publication venues, we prioritize journal papers over conferences and book chapters. In order to provide a timely and updated landscape of network visualization, we exclusively focus on publications ranging from **2000 to 2021**.

**Surveys** represent a practical and effective way to summarize and manage the great volume of published papers in a field [ML17]. In this category, we include papers with the following characteristics:

- **SC1**: They are a systematic review of literature about a specific network visualization discipline, providing a categorization of a broad selection of state‐of‐the‐art approaches;
- **SC2**: The focus of the survey is strongly oriented towards network visualization and has a clear contribution in this domain.

A non‐exhaustive list of the publications we include in our meta‐survey are the state‐of‐the‐art reports on the topic of general network visualization [HSS15, GFV13, CGZ*19], large [vLKS*11], dynamic [BBDW17], multi‐variate [NMSL19], multi‐layer [MGM*19] networks, group visualization [VBW17], and geospatial information in networks [SYPB21].

Computer graphics and graph theory surveys are considered out of scope. Brockenauer et al. [BC01] survey the literature for cluster and hierarchical graph drawings. Yu et al. [YFZ*20] review literature related to network motif discovery and their discussion focuses on a summary of the most popular algorithms to identify such patterns. While both papers are related to network visualization, their discussion is limited to discussing the algorithmic and complexity aspects, rather than their impact on visualization, not fulfilling **SC2**. Scientific visualization papers are also outside of our scope. Wang et al. [WT17] present a survey about graphs in scientific visualization; however, the focus is more on how graphs are used as a data structure to support the presented techniques rather than how they are used in the final presentation of the data to the user. We also exclude papers that focus only on interaction, such as the survey by Wybrow et al. [WEF*14] on interaction on multi‐variate networks.

**Task Taxonomies** enable preparing common and shared benchmark tasks that guarantee fair evaluations and comparisons between different visualization techniques [SYPB21]. The papers included in this meta‐survey and considered task taxonomies fulfill the following criteria:

- **TC1**: The paper provides a selection and categorization of possible tasks that can be applied for network visualization to extract information and/or insights;
- **TC2**: Taxonomies can be obtained empirically (e.g. through interviews or user studies) or by extending existing higher‐level ones and adapting them to support data types specific to a network visualization discipline;
- **TC3**: The taxonomies are evaluated, either empirically, or through a theoretical treatment.

Our literature research highlighted a scarce number of task taxonomies available for network visualization. We introduced requirements **TC2** and **TC3** to prioritize taxonomies of proven potential in supporting researchers. We include well‐established task taxonomies that support research in their own discipline, such as the papers by Lee et al. [LPP*06] on graph visualization, by Pretorius et al. [PPS14] on multi‐variate networks, and by Ahn et al. [APS14] on temporal networks. We also include task taxonomies suggested in the context of evaluations of visualization systems and design studies (**TC3**), such as the case of the work by Bach et al. [BPF14]. We exclude higher‐level visualization task taxonomies, such as the work by Valiati et al. [VPF06] on multi‐variate data visualization (**TC1**). However, these are often used as a basis for network‐oriented taxonomies: Pretorius et al. [PPS14] extend the Valiati taxonomy for multi‐variate network visualization (**TC2**). While out of our scope, we discuss them briefly in Section 6.1, and in our taxonomy coverage discussion (see Section 7.1). We also do not include task taxonomies proposed in the context of surveys [VBW17, NMSL19, MGM*19], as they are usually a collection and categorization of tasks gathered from the surveyed papers, therefore, not fulfilling **TC2** and **TC3**.

### 2.3 The roadmap categorization

To obtain our roadmap metaphor, we group surveys and task taxonomies into non‐overlapping sets based on their discipline (illustrated as large nodes with a heading and gray border in Figure 1).

**Figure 2:**
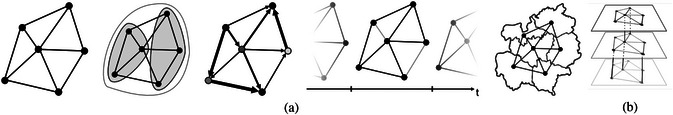
Illustrative examples. From left to right networks from the following network visualization disciplines: general, group structures, multi‐variate, dynamic, geospatial, and multi‐layer. Figure (a) courtesy of Hadlak et al. [HSS15], augmented with multi‐layer networks (b).

**Disciplines** refer to the branches of network visualization that deal with specific data types. While the networked nature of the data is common among all disciplines, each one is enriched with other attributes and dimensions, such as time (dynamic/temporal network visualization), geographic information (geospatial network visualization), facets, multiple layers, and multiple attributes. Each branch of network visualization has matured enough to be referred to as a network visualization discipline. A multitude of specific techniques were developed within each discipline to the point that it warranted the writing of one or more surveys, categorizing the corresponding approaches and summarizing the advancements and open challenges in literature (see Figure 2 for examples of network visualization disciplines identified in this meta‐survey). Summarizing, we consider a branch of network visualization to be a discipline if the following criteria are met:

- **DC1**: The branch of network visualization should have at least one survey discussing and categorizing techniques specifically designed for it (criterion of *maturity* and *variety*);
- **DC2**: The data type should have unique properties besides its networked nature (e.g. extra facets, time, multiple attributes or layers ‐ criterion of *specificity*).

Visualization of trees, for example, while being of great importance to the community and having more than one survey [WNF16, GK10] dedicated to it, is discussed under the general network visualization discipline, as their data type most closely resembles a simple networked structure.

Different disciplines can build upon a common theoretical ground onto diverging directions or one can be a specialization of another. We model these relationships as directed inheritance relationships depicting how the different disciplines are connected. In our roadmap metaphor, these can be seen as roads that lead from one network visualization discipline to the other. The green road signs indicate how data changes (i.e. with new features and, therefore, with added complexity) when travelling from one discipline to the other (see Figure 1). In the following, we present a high‐level discussion about the different network visualization disciplines that we have identified and discuss, how and why these are related to each other.

General network visualization entails visualization techniques devised for generic networked data. Therefore, it can be considered a generalization of all other disciplines. Large network visualization extends previous techniques for general networks to scale up to large numbers of nodes and edges, and it is shown accordingly in the green road sign in Figure 1. General network visualization can be extended by adding individual facets, which are further data attributes belonging to nodes, edges, or the entire graph itself, that have to be shown alongside the graph's topology. Depending on the type of facet, there are the disciplines of geospatial, multi‐variate, dynamic, or networks with group structures. Techniques devised for visualizing large groups are extensively discussed in the discipline of large network visualization (e.g. simplification by grouping, partitioning, or clustering) and can be leveraged in multi‐layer network visualization as well [MGM*19]. Visualization techniques for multi‐faceted networks inherit from all the disciplines dealing with individual facets as it focuses on those situations in which multiple facets have to be visualized at the same time. Furthermore, some facets can also be represented as different layers of a network which is represented in our roadmap as the multi‐layer visualization discipline.

### 2.4 Related work

As previously stated in the introduction, the inspiration for our work comes from the “Survey of Surveys” by McNabb and Laramee [ML17]. In their work, they draw a landscape of the information visualization literature through 86 survey papers, classified using the “Information Visualization Pipeline” by Card et al. [CMS99]. Further work on such meta‐surveys is proposed by Chatzimparmpas et al. [CMJK20], where they present a meta‐survey about the use of visualization in the interpretation of machine learning models, cataloging and discussing the surveyed papers, and proposing a meta‐analysis tailored for both newcomers and senior researchers in the field. Additionally, Alharbi and Laramee [AL18] present a meta‐survey about text visualization, cataloging each survey into one of five categories, obtained based on the main focus and themes found in each.

In our meta‐survey, we map the landscape of survey papers with a specific focus on the visualization of *graphs* and *networks* in more detail than McNabb and Laramee [ML17] and this work is, to our knowledge, the first comprehensive meta‐survey in network visualization. We also acknowledge the useful guide by McNabb and Laramee [ML19] about writing survey papers in visualization, which gave us suggestions used in the writing of this paper.

## 3 Surveys

In this section, we provide an in‐depth description of the content of selected survey papers, organized by discipline, and, within each discipline, we order the papers by publication date to suggest a sense of progression over time. We begin each subsection with a short definition of the specific discipline as depicted in our roadmap (see Figure 1). To ease the discussion, some less relevant papers in our scope are referenced and shortly discussed at the beginning of each subsection to further provide background and context (see Table 2). Conversely, in‐depth surveys are discussed within their respective discipline using a structured summary and visual cues, to ease reading and comprehension, as follows. The summary is divided in: *motivation* (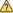), which also includes the goals of the survey; *contribution* (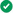), which presents an outline of the proposed categorization; and *open challenges* and future work (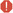).

**Table 2:** Table reporting the surveys in this paper arranged by discipline (see Section 2.2) and chronological order. References highlighted in pink represent literature we reference and include in our discussion but do not describe in detail in Section 3. All included surveys are part of our terminology consolidation (see Section 5).

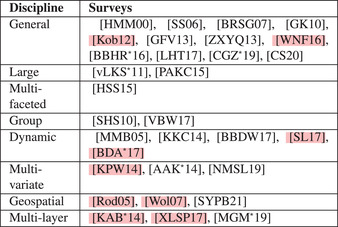

### 3.1 General network visualization

A graph G=(V,E) is a data structure composed of a set of vertices $V=\{v_{1},\text{…},v_{n}\}$ (nodes) and a set of edges that are (un‐)ordered pairs of vertices $e=(v_{i},v_{j})\in E\subseteq V\times V$ representing their relationships (links). *General* network visualization refers to techniques that tackle the problem of visualizing simple networked data, typically focusing on how to produce a layout for the graph [BBHR*16, CS20], improving visual scalability of the edges [ZXYQ13], graph drawing aesthetic criteria [BRSG07], and visualization of trees [GK10]. While in our roadmap (see Section 2.3), all these heterogeneous topics fall into this discipline, to ease reading and comprehension, we further categorize the surveys into different branches.

#### High‐level network visualization

■ **Herman et al**. [HMM00] publish one of the first surveys on graph visualization with a perspective more oriented towards information visualization rather than traditional graph drawing.


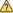
 *Motivation*: In the words of the authors, “*the traditional concerns of Graph Drawing become less relevant in graph visualization*” [HMM00, p. 6], since the latter deals with much larger, real‐life graphs.


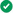
 *Contribution*: In their survey, the authors review papers regarding (i) graph layout, (ii) graph interaction and navigation, and (iii) complexity reduction. Issues and limitations regarding scalability (both computational and visual) are discussed. Concerning the first category the survey presents an overview of traditional layout techniques outlining the most widely adopted layout approaches: tree‐based, layered (as in the work by Sugiyama [STT81]), and energy‐based/force‐directed layouts. In the second category, the authors present navigation and interaction approaches for graph visualization. The interaction and navigation techniques are classified as zoom and pan approaches (i.e. geometric and semantic zooming), focus+context approaches (i.e. fisheye and focus+context layout techniques), and incremental exploration and navigation (i.e. details‐on‐demand). Complexity reduction methods, that is, solutions to simplify the graph to improve scalability, are discussed (e.g. 3‐D representations, layout with alternative geometries, clustering methods). The survey also presents a review of available network visualization systems present at the time of publication.


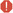
 *Open Challenges*: In terms of future work, the authors outline the need for more focus on cognitive aspects of graph visualization techniques, further research on focus+context layout techniques, and more attention should be given to clustering and aggregation to reduce the number of visual elements being displayed.

■ **Schulz and Schumann** [SS06] present a generalized view of graph visualization.


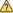
 *Motivation*: The survey provides a systematic overview of the problem by combining two perspectives on graph visualization: the one from the graph drawing community, more focused on optimized layouts and node‐link representations, and the second from the information visualization community, more focused on larger (hierarchical) structures, multiple views, and interactivity.


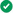
 *Contribution*: The authors divide the surveyed visualization techniques as follows: (i) hierarchy and (ii) general network representations. Hierarchy representations are further divided into explicit and implicit (inclusion relationships are explicitly shown), axes‐oriented, and radial (fixed arrangements of the hierarchy levels either line‐by‐line or in concentric circumferences). General network representations are categorized similarly, into explicit and implicit, directed and undirected (whether the vertex pairs are ordered or not), free layouts (i.e. force‐directed layout), styled (i.e. a grid layout), or fixed where node and edge positions are constrained (i.e. flight route map and geospatial networks).


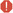
 *Open Challenges*: The survey provides indications and general practices on which of the presented techniques to choose depending on the user requirements or data characteristics, concepts that became part of a network visualization framework [SNS06]. The paper concludes by suggesting extensions to this work, in particular, to support hypergraphs.

■ **Chen et al**. [CGZ*19] explore visualization for association relationships in static graph data.


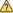
 *Motivation*: Exploring relationships in complex datasets is a challenging task: advancements in visualization approaches that combine graph drawing theory and human intelligence have the potential to support users in finding insights in these data. This paper aims at providing a unified model for visual analysis of networked data, that would encompass topics from graph simplification and visualization to interaction.


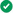
 *Contribution*: The survey proposes a visual analysis model for networked data, comprised of three stages: (i) relationship modelling, (ii) visualization techniques, and (iii) graph simplification and interaction. In the first stage, algorithmic pre‐processing for relationship extraction adopting a combination of graph mining, data modelling, and graph analysis is discussed. Graph analysis tasks are also reported from the work of Pretorius et al. [PPS14] and Lee et al. [LPP*06]. The second stage discusses visual techniques to support an intuitive and effective representation of the graph data, including node‐link representations, adjacency matrices, hypergraphs, flow diagrams, graphs with geospatial information, multi‐attribute graphs, and space‐filling visualization techniques. The last stage surveys techniques such as graph filtering, node clustering, edge bundling, dimensionality reduction for graph data, and topology‐based graph transformation.


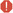
 *Open Challenges*: Major research trends are discussed as directions for future work, specifically, augmenting visualization technology with machine learning for pattern mining and discovery, improving node clustering to tackle visual clutter for large graphs, multi‐view solutions for collaborative analysis, and leveraging 3‐D and virtual reality technologies for network visualization.

#### Node‐link layouts and aesthetics

■ **Bennet et al**. [BRSG07] focus on the aesthetic principles behind node‐link network representations.


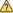
 *Motivation*: The aesthetic principles were introduced to provide a set of known and (empirically) tested layout criteria to improve the readability of such diagrams (e.g. limiting the number of edge crossings [BMRW98, TR05]). The authors collect findings concerning which criteria fit best for each application.


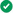
 *Contribution*: The authors survey papers that discuss layout aesthetic heuristics, first limited to the appearance of nodes and edges, then applied to the entire graph layout, and finally concerning domain‐specific applications. The surveyed heuristics are grouped into (i) node and (ii) edge placement, (iii) overall graph layout, and (iv) domain‐specific (e.g. UML diagrams or social network diagrams) aesthetics.


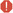
 *Open Challenges*: The survey outlines that little work has been done in applying aesthetic heuristics for graph visualization from other fields (e.g. cartography, graphical design). Aesthetic criteria can also conflict with each other: in this context, evaluating the (application‐specific) trade‐offs between different metrics can provide valuable design insights. Finally, more evaluations need to be performed on readable graph layouts to understand the perceptual basis of these heuristics.

■ **Gibson et al**. [GFV13] survey 2‐D node‐link graph layout techniques for information visualization.


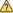
 *Motivation*: The problem of computing a layout of a graph is still a challenge, even after 50 years after Tutte's barycentre method [Tut63]. This process has the burden to provide visual aid to the analysis and support the understanding of the graph, possibly highlighting structures and patterns in the data, necessary to discover insights [HFM07].


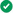
 *Contribution*: The paper analyses 30 years of research oriented towards graph layouts. The categorization proposed by the authors divides layout algorithms into three families: (i) force‐directed (energy‐based), (ii) algorithmic approaches (dimensionality reduction), and (iii) multi‐level force‐directed. In the same context, the survey discusses common aesthetic criteria, also referencing the work by Bennet et al. [BRSG07], and evaluation methods for these techniques. Multi‐level approaches are discussed as computational improvements to address the limited scalability of traditional force‐directed techniques. The paper also discusses how node attributes (especially in multi‐variate or multi‐faceted graphs) could be utilized in the graph layout process.


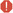
 *Open Challenges*: The survey outlines the current problems with validating graph layout techniques. One rather fascinating discussion concerns how there is no explicitly known relationship between drawing principles, layout, and user tasks [DS09, SGF11]. Purchase [Pur00] showed that while the effect of individual aesthetics was undoubtedly significant in graph exploration tasks, the choice of (force‐directed) layout method did not largely affect user performance on those same tasks. Even with the introduction of multi‐level algorithms, visual scalability remains a challenge, as layout quality does not appear to improve significantly over spring embedder techniques [Ead84, FR91], except for regular structures (e.g. meshes). This suggests that the drawing principles for small graphs might be different from the ones that could benefit larger graphs, and, therefore, other approaches that highlight other structural properties of the graph should be considered (such as LinLog [Noa07]) as well as layout methods that make use of node or edge attributes.

**Figure 3:**
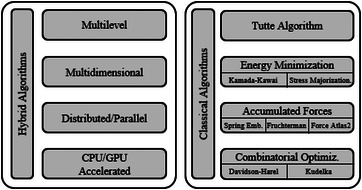
The taxonomy of force‐directed algorithms presented by Cheong et al. [CS20]. Classical spring‐embedder approaches fall into the “Accumulated Forces” category.


■ **Cheong et al**. [CS20] aim at providing a comprehensive summary of the last 50 years of research on force‐directed algorithms in graph drawing (see Figure 3).

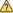
 *Motivation*: As force‐directed algorithms have been developed for the last 50 years, they have found their way in several application domains. The survey addresses the recent developments and provides a summary of state‐of‐the‐art force‐directed algorithms in schematic drawings (i.e. representation of elements of a network using simple schematic symbols) and node placement.


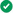
 *Contribution*: First, the survey presents a comprehensive description of all typologies of force‐directed algorithms, divided into (i) classical force‐directed algorithms (which include the methodologies presented in previous surveys [HMM00, GFV13]), and (ii) hybrid approaches, specifically pointing at advanced computing methodologies to speed up the layout computation process (i.e. multi‐level approaches and parallel, distributed, and GPU implementations). Second, the survey lists and categorizes papers according to the application domain where force‐directed approaches were adopted. These are (i) aesthetic drawings for general networks, (ii) component placement and scheduling in high‐level synthesis of VLSI circuit design, (iii) information visualization, (iv) biological network visualization, and (v) node placement and localization in sensor networks.


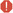
 *Open Challenges*: While the survey does not clearly outline future work opportunities, the scope of the paper is to establish a taxonomy of the plethora of force‐directed techniques which have been presented over the years and introduce them as valuable tools to solve a wide variety of visualization problems in several application domains.

#### Edge bundling

■ **Zhou et al**. [ZXYQ13] survey the topic of edge bundling which is a common technique in information visualization to reduce visual clutter occurring with the depiction of a large number of edges.


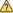
 *Motivation*: Different from graph simplification techniques such as clustering, filtering, and sampling [ED07] , edges are deformed and grouped into bundles. The surveyed techniques are presented in the context of remediating the visual clutter problem on graphs, parallel coordinate plots, and flow maps.


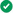
 *Contribution*: The presented taxonomy includes three edge‐bundling categories: cost‐based (e.g. ink [PNK10] or energy [HVW09] minimization), geometry‐based, and image‐based techniques. In the second category, bundling is computed along a geometry that is either derived from the hierarchical clustering of the graph [Hol06] or a grid [QZW06] (when such a clustering is not present). Image‐based edge bundling approaches are also discussed, which require the edge clusters to be computed beforehand and then rendered using image‐enhancement tools (e.g. silhouettes, shadows, halo effects, faded histograms, and splatting).


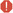
 *Open Challenges*: The authors of the survey identify notable directions for future work, such as the readability of the bundled diagrams, algorithm complexity, interactions (i.e. intuitive navigation), and research on geometry‐based bundling techniques. Moreover, the survey points out that only a few works present a systematic evaluation of edge bundling approaches.

■ **Lhuillier et al**. [LHT17] expand the previous survey by Zhou et al. [ZXYQ13] on edge bundling.


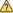
 *Motivation*: The survey by Zhou et al. [ZXYQ13] did not cover some critical elements for understanding bundling (e.g. bundling attributed and time‐oriented data, interaction techniques). This survey aims at introducing comprehensive taxonomy of bundling methods, a framework that would precisely define what bundling *is*, and a formal platform for comparing the different solutions from a technical perspective.


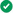
 *Contribution*: The survey introduces a comprehensive taxonomy of bundling methods for both graphs and trail sets (i.e. oriented curves in $R^{d}$, usually used to describe the motion of objects in space), a framework for comparing these algorithms from a technical perspective, and an extended discussion on what bundling is, which tasks it supports, and how it compares with other simplification techniques. The design space of edge bundling for graph drawings is divided into techniques for static and dynamic graphs. The survey categorizes papers that propose edge bundling techniques for (i) hierarchical and compound graphs, (ii) directed graphs, flow maps, (iii) confluent drawings, and (iv) 3‐D graph visualization techniques. For trail‐sets, the survey proposes a similar classification as the one proposed for edge bundling, dividing the techniques into static and dynamic trail‐sets.


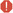
 *Open Challenges*: As there are no objective or well‐defined criteria to determine what a good bundling is, research is required on a standardized assessment of the visual quality of the bundling. Moreover, bundling faithfulness, defined as the amount of information lost or altered by the bundling process, is a concept that is typically overlooked when discussing bundling techniques. Finally, user‐controlled edge bundling is another hot topic in the field (i.e. parameter tuning and interactive bundling).

#### Matrix reordering

**Figure 4:**
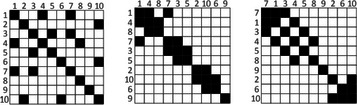
Different matrix reorderings can reveal different patterns in network topology. Courtesy of Behirsch et al. [BBHR*16].


■ **Behirsch et al**. [BBHR*16] survey algorithms that reorder the rows and columns of adjacency matrices or tabular representations of networks.


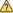
 *Motivation*: Adjacency matrix reordering (i.e. finding a proper order for the rows and columns comprising a visual matrix) has been historically done manually. Recently, several automatic methods have been presented, which guarantee fast results and can highlight clusters and higher‐order patterns (see Figure 4).

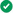
 *Contribution*: The authors provide a comprehensive overview of matrix reordering algorithms that are widely used in different fields, e.g. statistics, bioinformatics, and graph theory. The surveyed techniques are grouped into seven algorithmic families and the classification of each is tailored toward the goal of providing guidance on which algorithm to employ based on the size and structure of the data. The categories are the following: (i) statistical (Robinsonian) approaches, (ii) spectral methods, (iii) dimension reduction, (iv) heuristic approaches, (v) graph‐theoretic approaches, (vi) bi‐clustering algorithms, and (vii) interactive and user‐controlled approaches. The survey also presents suggestions and guidelines on which algorithm to select in typical usage scenarios, based on the authors' observations, analysis, and empirical knowledge.


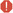
 *Open Challenges*: Improving on global and local algorithms, leveraging human and computer skills in interactive approaches, and developing quantitative measures to evaluate the quality of the patterns resulting from the ordering is outlined as the most prominent directions for future research. Hybrid graph representation approaches (see, e.g. [HFM07, ADM*19]) are also pointed out as an interesting direction.

#### Tree visualization

■ **Graham et al**. [GK10] survey the topic of multiple tree visualization, that is the logical structures formed by merging multiple trees.


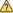
 *Motivation*: Multiple tree visualization is relevant in several application domains. Tree visualization is a prominent field in graph drawing; however, multiple tree visualization has not received as much attention. Therefore, this motivated the authors to gather and categorize visualization research done in this field.


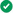
 *Contribution*: First, single tree visualization techniques are discussed, as in the survey by Wang et al. [WNF16] on 2‐D tree visualization, and this mostly covers well‐known approaches (node‐link, radial, etc.). Building on that, the survey presents visualization techniques for the combination of two trees. In this context, papers are categorized as to whether they resort to (i) linking separate tree representations, (ii) sharing the colour space between multiple trees, (iii) use animation (temporal separation), (iv) matrix comparison, or (v) spatial agglomeration of the two tree structures. Multiple tree visualizations mostly extend the previous approaches, and the survey applies the same categorization – with the addition of 3‐D and atomic representations. Atomic representations show the individual trees in the collection as atomic items in a details‐on‐demand fashion designed for very large collections of trees. The survey also collects high‐level tasks for multiple tree visualization.


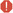
 *Open Challenges*: Future work directions include focusing on more conclusive user studies on this topic, as the current ones had a narrow scope or few participants. Moreover, as more and more trees are merged together, the complexity of the resulting structure presents a significant challenge, with layered graph drawing being considered as a potential starting point to address this challenge.

### 3.2 Large network visualization

*Large* network visualization extends the classical general network visualization techniques to accommodate a much larger number of nodes and edges. The size of graphs of commercial and scientific interest has grown exponentially over the years: networks with thousands of nodes and edges were considered to be large in the early 2000s, with recent research tackling graphs with millions of nodes and edges [Hu05, HJ07, ADLM18]. Large networks can also leverage aggregations, such as grouping strategies that simplify and represent a more abstract view of these networks, which is why it is also connected to group network visualization (see Figure 1).

■ **Von Landesberger et al**. [vLKS*11] describe the field of large network visualization according to the information visualization reference model by Card et al. [CMS99].


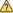
 *Motivation*: The visual analysis of large graphs is gathering more and more attention, as we enter the big data era. Effective analysis requires more than just visualization, and this survey aims at reviewing a range of techniques including algorithmic analysis, visualization, and interaction, that together form the basis of effective visual exploration of large graphs.


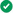
 *Contribution*: The pipeline is comprised of four stages: (i) algorithmic pre‐processing (for simplifying the network structure and removing noise), (ii) visual representation, (iii) user interaction, and (iv) visual analysis. The research surveyed in this paper is discussed on a per‐stage basis. Algorithmic pre‐processing surveys techniques for graph complexity reduction, such as graph filtering and aggregation. Visual representation discusses visualization approaches for a broad range of graph types, with a distinction between static and dynamic graphs, preceding the categorization by Beck et al. [BBDW17]. User interactions are categorized based on the stages of the information visualization reference model of Card et al. [CMS99] , depending on whether the user affects the current view, the displayed data, or their visual abstraction. View interactions entail panning and zooming or the use of magic lenses, which locally distort the data visualization on a single focus or multiple foci point(s) [EDG*08]. Visual abstraction interactions include highlighting, brushing, and semantic zooming, as in an overview + details‐on‐demand approach [EDG*08, WWZ*18]. Interactions on the data include filtering and changing graph aggregations. Concerning the fourth and final stage, graph analysis, its techniques are categorized depending on whether the target of the analysis is graph structure (e.g. identification of important nodes) or the examination of similarities and differences between multiple graphs.


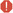
 *Open Challenges*: Discussion on future work is divided into graph visualization and interaction, visual analysis systems, and conceptual issues. Concerning visualization, for large graphs tackling visual scalability represents one of its main challenges. This could be addressed through a more visual analytics‐oriented approach, where users can be increasingly involved in the graph layout process to improve its readability, as with the use of interactive filtering and aggregation techniques. Another challenge lies in the growing number of different graph types, from more known and studied categories, such as dynamic graphs to compound graphs and hypergraphs. Uncertainty is another emerging challenge in this discipline, as it has been proven how it affects analytical decisions [GS*06]. Uncertainty for node and edge attributes might be conveyed by using techniques from multi‐variate data visualization, while visualization for structural uncertainty is still in its infancy, with few approaches dedicated to this topic. Perception and graph interaction are also outlined as important scientific challenges in the field of large graph visualization. Finally, the survey discusses that appropriate evaluation methods for graph analytics are still an argument of debate in the community, to surpass traditional metrics such as completion time and error rates.

■ **Pienta et al**. [PAKC15] survey graph exploration and visualization techniques in the context of sense‐making for large graphs.


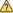
 *Motivation*: The term *graph sense‐making* refers to the iterative process of understanding graph data. It is a complex and abstract task that depends on both domain and data. This survey aims at reviewing graph sense‐making literature focusing on scalability and interaction techniques that support such cognitive processes.


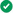
 *Contribution*: The authors present a graph sense‐making hierarchy, which they also use to categorize the different techniques. This hierarchy identifies two main sense‐making paradigms: (i) global and (ii) local views, which broadly represent top‐down and bottom‐up exploration. Local view approaches are further divided into targeted (where the user has a data‐centric goal) and free (or open‐ended) discovery. The papers surveyed in this review were chosen and discussed to understand their potential in supporting a scalable sense‐making process. Global view approaches include techniques based on filtering, sampling, partitioning, and clustering of the data. Free discovery includes graph exploration and network *motifs* (i.e. patterns of subgraphs which appear unusually often in a network [MSOI*02]), with targeted discovery including subgraph pattern matching and navigation techniques (i.e. exploration with a known objective or destination).


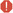
 *Open Challenges*: The authors argue that most techniques are extremely expensive from a computational standpoint, outlining opportunities for techniques based on approximated solutions (i.e. heuristics) that can be used in an interactive environment. For subgraph matching, future work includes further investigation on both visual query construction and results representation. Moreover, it is discussed how network motifs could be used to improve the visual scalability of graph representations. Finally, multi‐touch approaches and gestures, nowadays extremely common, could represent an opportunity to create innovative exploration methods but present also several challenges, such as which gestures are appropriate for each task and graph type. Research on these advanced interaction techniques has recently obtained significant interest [TSS18, SSS20].

### 3.3 Group network visualization

*Group* network visualization is defined as a graph G=(V,E), where group structures are a family of subsets of vertices $S=\{S_{1},\text{…},S_{k}\}$, with each $S_{i}\subseteq V$ [VBW17]. Basic definitions include disjoint or overlapping groups, whether a vertex can only belong to one set or many, and unstructured (flat) or structured, depending on if the grouping further defines a hierarchy between members of the same group. Group network visualization is strongly related to both large network visualization, utilizing grouping as an aggregation strategy, and, multi‐layer network visualization as a way to group multiple layers together or to present different groups within each layer (see Figure 1). Furthermore, it is also defined as a facet in the scope of multi‐faceted network visualization [HSS15] that is depicted alongside other facets (i.e. time or multiple attributes).

■ **Schulz et al**. [SHS10] survey the topic of implicit hierarchy visualization, where the connections between the elements of the hierarchy are not explicitly represented, as, for example, in Treemaps [Shn92] and Icicle Plots [KL83].


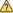
 *Motivation*: There exists a robust body of knowledge about implicit group visualization techniques, inspired by more than 25 years of research. Instead of directly exploring this plethora of techniques, the paper identifies four dimensions based on common visualization principles in the discipline. The aim of the paper is to present a design space, built along these dimensions, comprehensive enough to classify the existing techniques but also able to suggest and explore new ones.


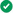
 *Contribution*: This survey describes a design space with four dimensions: (i) design dimensionality (2‐D or 3‐D), (ii) graphical primitives for node representation, (iii) edge representation (through inclusion, overlap, and adjacency), and (iv) layout (space subdivision and packing). Other than using it to survey the existing techniques, the paper presents an implementation of the proposed design space, to enable access and ease of experimenting with new design combinations.


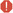
 *Open Challenges*: Summarizing, the survey discusses promising opportunities for further research, including, investigating subtler design aspects, such as parametrization and mixing of the design choices and layout techniques. These innovative combinations could yield unique visual representations that are more effective in expressing the hierarchical characteristics of the data.

**Figure 5:**
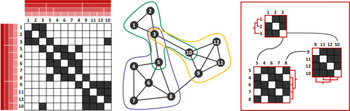
Groups in networks. From left to right, attached juxtaposition (hierarchy as red bars), superimposed (contouring), and embedded (hybrid approach). Courtesy of Vehlow et al. [VBW17].


■ **Vehlow et al**. [VBW17] in their survey propose a taxonomy focused on explicit visualizations of group structures in networks, complementing the survey by Schulz et al. [SHS10].


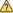
 *Motivation*: The paper describes a two‐layered design space and uses it to classify a large number (110) of group visualization techniques.


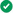
 *Contribution*: The proposed categorization has two dimensions: (i) the group structure and (ii) the group visualization taxonomy. The group structure is divided into four classes, depending on the structure itself (flat or hierarchical), and whether overlapping between sets is allowed or not. Specific algorithms also present either *crisp* (one element fully belongs to one or more groups) or *fuzzy* groupings (one element can belong to different sets to different extents). The taxonomy of the group visual encodings includes (see Figure 5): (i) use of visual node attributes (colour, glyphs), (ii) juxtaposition (separate, attached), (iii) superimposition (line overlay, contour overlay, partitioning), and (iv) embedded (node‐link, hybrid). The survey also discusses approaches that deal with dynamic graphs having both static and dynamic groupings. Concerning edge group visualization (which can be defined in the same way as groups of vertices), the survey proposes a similar visual encoding taxonomy, with visual edge attributes, juxtaposition, superimposition, and embedded visualization techniques.

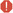
 *Open Challenges*: The survey points out how group structure visualization is only partially supported by general tasks in existing taxonomies (see Table 3 in Section 7.1). The survey, therefore, proposes a task taxonomy for the discipline by collecting tasks from existing technique and evaluation papers to support a future generation of a taxonomy specific for this discipline. Moreover, the visualization of dynamic graphs and groupings is an under‐investigated direction. While some initial attempts have been made, such as in the work of Vehlow et al. [VBAW15] for time‐varying groups in a dynamic graph, they only cover disjoint flat or hierarchical group structure, leaving gaps in the proposed design space. Visual analytics methods could be a promising research direction to tackle overlapping and fuzzy groupings in larger graphs. These approaches combine data mining techniques with the visualization of group structures in an interactive approach.

### 3.4 Dynamic network visualization

*Dynamic* network visualization is defined as the discipline that investigates the visualization of networks that change over time. Traditionally, dynamic networks are described as a series of timeslices, that is graph evolution is portrayed as a sequence of graph snapshots, one for each time unit [BBDW17]. Dynamic network visualization can be considered by adding the time (temporal) facet to approaches for general network visualization. The temporal facet is also considered in multi‐faceted network visualization alongside other facets. In the scope of multi‐layer network visualization, the temporal information associated with a graph can be represented as a set of layers (one for each time slice), modelling the dynamics or evolution of a network (see Figure 1). Shaobo et al. [SL17] present a survey of available tools and technologies available for the visualization of dynamic networks.

■ **Moody et al**. [MMB05] survey graph and visualization principles, in the context of temporal representations of social networks.


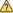
 *Motivation*: The goal of the paper is to investigate how the changes and temporal developments of a network can be reflected in its graphical form. Specifically, the paper addresses theoretical questions about temporal representations of social networks and how to present the changes to the user.


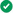
 *Contribution*: The authors categorize papers based on the representation of time, either as (i) discrete (using timeslices) or (ii) continuous. In discrete time analysts can focus on identifying changes from one network state to the other. To visualize continuous network data the authors characterize a network by using a time window that spans an interval aggregating events within it, which can either be overlapping (i.e. a moving average) or non‐overlapping (i.e. time windows are separate or distinct). Based on the encoding of time, the authors survey graph layout algorithms categorizing them in two classes, namely, static flipbooks and dynamic network movies. In flip books, network dynamics are represented as appearing and disappearing social relationships, with each “page” representing a timeslice. In dynamic network movies, nodes move freely through time and update the resulting layout according to the social‐relational changes that happen in the network similar to event‐based networks (see, e.g. [SAK18, AMA22]).


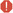
 *Open Challenges*: The authors suggest extending dynamic network movies from an exploratory data analysis stage to a confirmatory analytic modelling stage, by linking dynamic network movies to statistical models describing the network's change. A closer connection between the two would support building better statistical tools to model dynamic network changes.

■ **Kerracher et al**. [KKC14] map the design space of temporal graph visualization.


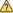
 *Motivation*: This paper presents a wider look at visualization techniques to represent temporal graph data, considering the conceptual tasks required to make sense of graph changes over time.


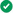
 *Contribution*: The survey identifies two independent dimensions for temporal graph visualization: (i) the graph structural and (ii) temporal encoding. The former describes how to represent the graph structure, with the most common options being space‐filling, node‐link, and matrix approaches. Based on Javed and Elmqvist's [JE12] design patterns and Gleicher et al.'s [GAW*11] comparative designs, this survey identifies seven temporal encoding strategies, including sequential views, juxtaposition, additional spatial dimension, superimposition, merged views, nested views, and time as a node in the graph.


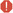
 *Open Challenges*: The authors suggest investigating further the use of adjacency matrices and space‐filling techniques, specifically, in the context of dynamic network representations. Furthermore, they also identify juxtaposition and sequential views as being the more common options when encoding temporal data in networks and encourage the investigation of less popular approaches from their proposed design space.

**Figure 6:**
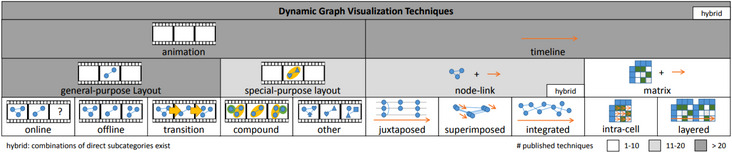
The design space of dynamic network visualization broadly categorizes approaches as “time‐to‐time” (animation) or “time‐to‐space” (timeline). As the brightness of the table background encodes the number of techniques, it is possible to evaluate at a glance more or less saturated categories. Courtesy of Beck et al. [BBDW17].


■ **Beck et al**. [BBDW17] present one of the most influential and comprehensive characterizations of the dynamic network visualization discipline.


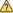
 *Motivation*: With the increased availability of time‐varying network data, dynamic network visualization quickly became a mature and thriving research field. As more various and novel approaches continuously developed, a clear need for a comprehensive review emerged. This survey aims at addressing this gap in visualization literature.


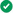
 *Contribution*: The authors collected and tagged 162 publications, categorizing them by the type of publication, the visual representation of time, the visualization paradigm, the type of evaluation that was conducted, and the target application domain. This multi‐level taxonomy has become the standard way to categorize technique papers related to this discipline (see Figure 6). It divides the design space into (i) animation‐ (time‐to‐time mapping) and (ii) timeline‐based (time‐to‐space mapping) techniques. Only very few of the surveyed approaches (e.g. Animatrix [RM14]), adopt a matrix‐based representation of the network. A section of the survey categorizes evaluation papers, dividing them into evaluation frameworks, algorithmic evaluations, and user studies. Specifically, in the first category, task taxonomies and papers related to aesthetic criteria are discussed, as they are the foundations for fair and reproducible user and experimental studies.

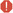
 *Open Challenges*: First, there is a lack of guidelines and direction regarding which technique to choose based on the application. Second, investigating the effect of a user's cognitive load when watching animations [BLIC20, LPP*21] is an open challenge. Moreover, visual scalability is still a major concern in the design of most dynamic network visualization methods. Hybrid combinations of timeline and animation approaches are under‐explored. Finally, multiple (dynamic) data dimensions, interaction methods, and continuous (event‐based) representations are also a topic that presents research directions with significant potential for this discipline.

### 3.5 Multi‐variate network visualization

*Multi‐variate* networks are defined by Kerren et al. [KPW14] as an underlying graph *G* plus *n* additional attributes attached to the nodes and/or edges. The main challenge in visualizing multi‐variate networks is showing both the underlying network topology and its associated attributes at the same time [NMSL19]. Multi‐variate network visualization is the result of augmenting general network visualization techniques with *n* additional variables connected to the nodes and/or edges of the graph. It is also considered a facet by the multi‐faceted network visualization discipline, typically represented alongside other facets. In the context of multi‐layer networks, the multi‐variate aspect is also discussed, as a way to model each attribute of the network as distinct layers (see Figure 1).

■ **Archambault et al**. [AAK*14] formalize the concept of multi‐variate temporal networks, which, in terms of our categorization, is borderline between dynamic and multi‐variate visualization; however, the focus is leaning more on the co‐representation of time with other dimensions.


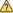
 *Motivation*: While time can be considered as yet another dimension in a multi‐variate network, it is perceived differently by humans. Therefore, this fact should be exploited when time is represented alongside other dimensions. The paper, therefore, defines, characterizes, and summarizes the data and visualization techniques related to this field, outlining the use of these techniques in software engineering and other application domains.


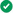
 *Contribution*: Time is introduced as a further dimension, that can both affect the topology of the network and the state of all other attributes. The papers surveyed cover aspects specific to static and dynamic graph visualization and analytics. The survey analyzes the relationship between the discussed temporal multi‐variate network framework with respect to the problems coming from software engineering, including aspects like data size, attributes, dynamics, and time modelling. The survey also covers orthogonal concepts from static and dynamic network visualization and analytics, such as static layout algorithms and dynamic network visualization.


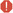
 *Open Challenges*: The first challenge identified by the survey is related to attribute dimensionality. Existing visualization techniques can show up to three attributes per graph element (e.g. shape, size, texture, colour, and shading). However, it can easily get cluttered as the size of the graph increases, compromising the readability. Parallel coordinate plots and dimensionality reduction methods are discussed as ways to address this challenge; however, no methods fully enable users to correlate the underlying graph structure to its attributes and between the attributes themselves. The second challenge discussed is capturing patterns. The sole depiction of the graph's changes is often insufficient compared to the importance of locating patterns in a multi‐variate graph. The third, and last, challenge identified in the survey is scalability with respect to the graph's size and its associated variables.

**Figure 7:**
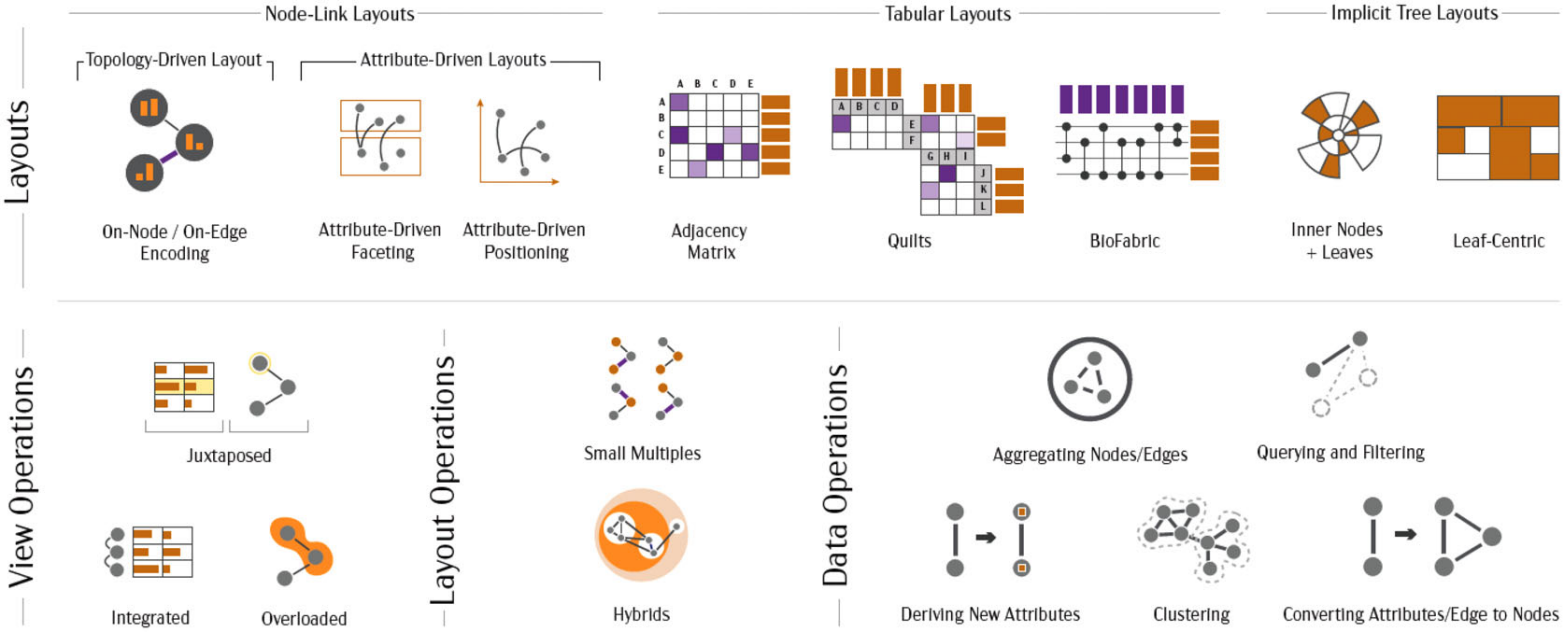
Typology of layouts and operations in multivariate network visualization as presented by Nobre et al. [NMSL19]. Approaches are classified according to the layout and operations supporting the views, layout, or data. Courtesy of Nobre et al. [NMSL19].


■ **Nobre et al**. [NMSL19] survey papers on multi‐variate network visualization.


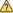
 *Motivation*: Most real‐world networks have attributes that characterize their nodes or edges. When the topology of the network has to be displayed *along* these extra attributes, several challenges arise. The developed techniques tackle these differently: the paper introduces new typologies for multi‐variate network tasks and visualization methods, surveying the literature to classify them according to their formal typology.


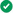
 *Contribution*: The survey includes 210 publications on multi‐variate network visualization and reports a typology of tasks, visualization techniques, and evaluation methods for multi‐variate network visualization. The presented typology of tasks inherits and simplifies the one from Pretorius et al. [PPS14] (see Section 6), and its purpose in the scope of the survey is to characterize and recommend techniques based on their suitability for specific tasks. The paper categorizes the surveyed techniques by layout (further divided into node‐link, tabular, and implicit) and by the supported operations, specifically, if they act on the views, layout, or data (such as aggregation or querying) (see Figure 7). The survey also provides explicit guidelines and recommendations for the usage of each technique, considering the network and attribute types. The evaluation methods used throughout the corpus of surveyed papers are discussed, outlining that the majority of techniques were evaluated through use cases (i.e. informal evaluations without any quantitative measure of the tool's validity), followed by controlled experiments, and user and usability studies.

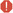
 *Open Challenges*: While node‐link techniques have received a lot of attention, tabular techniques (i.e. adjacency matrices) present significant potential for multi‐variate network visualization. Similarly, a limited number of techniques investigate the visualization of edge attributes: tabular and integrated approaches could be potential areas where this challenge can be tackled [SSE16, VABK20]. The evaluation analysis shows that very few studies rigorously investigated the trade‐offs and benefits of different multi‐variate visualization techniques – task taxonomies have the potential and purpose to drive more formal evaluations.

### 3.6 Geospatial network visualization

*Geospatial* networks are a subgroup of spatial networks whose nodes and links can be associated with geographic locations either on Earth or other planets [SYPB21]. These are among the most commonly known network visualizations: they are used, for example to portray trade and financial connections between countries and regions [ATL*22] or to display flight connections [Rod05]. Layout and visualization of metro maps, which can be seen in every subway station, is a thriving field of research (see, e.g. [HMdN06, Wol07, WC11, Nöl14, Wu16]). However, it does not fit this discipline as metro map layout is more focused on the topology of the underlying network rather than its geographical information. Geospatial networks are considered to be specialized in representing the geographical aspect of the data discussed in multi‐faceted network visualization (see Figure 1).

**Figure 8:**
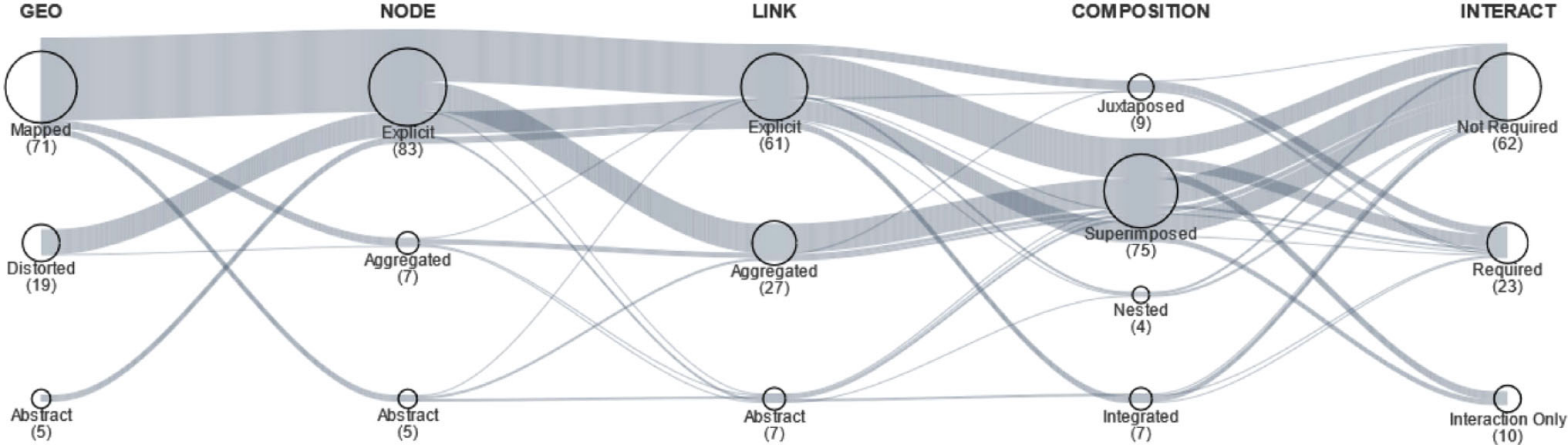
The design space for geospatial networks as presented by Schöttler et al. [SYPB21]. The dimensions include representation of the geographical facet, as well as, of nodes and edges, composition technique, and use of interaction. Lines represent techniques that are aggregated into bands. The number of techniques is encoded in the circle width and shown in brackets. Courtesy of Schöttler et al. [SYPB21].


■ **Schöttler et al**. [SYPB21] present a systematic review of geospatial network visualization approaches.


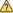
 *Motivation*: Numerous techniques have been devised for the visualization of networks with a geographical facet. The survey aims at providing a structured review of these techniques by proposing a consolidation of the different terminologies used and establishing a design space that would support designers in building a balanced and purposeful visualization for this type of networked data.


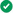
 *Contribution*: The dimensions of the proposed design space are as follows: (i) geographical facet representation, (ii) network representation (for both nodes and edges), (iii) composition (how the topology and geography are combined in the visualization), and (iv) use of interaction (see Figure 8). The survey spans 95 publications and outlines a design space for visualizing and interacting with geospatial networks. The geographical representation is concerned with how geospatial information is encoded and ranges from explicit (representations that use a cartographic map), to distorted (representations that use displacement of spatial positions according to some property of the network), to abstract (representations that use encodings not based on map projections). The categorization has several overlaps with the multi‐faceted visualization survey by Hadlak [HSS15], in the section about the geographical facet. It is noteworthy how the interaction is discussed, that is to what extent a technique requires user input for exploring the visualization. It can either be (i) not required, (ii) required, or (iii) an interaction technique in its own right (i.e. the scope of a paper in this category is not a visualization, but rather an interaction technique).

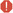
 *Open Challenges*: Geospatial network visualization shares several similarities with multi‐variate visualization, which can be used to design specific visualizations for this field. Handling co‐located nodes (a challenge specific to nodes representing locations) and link density are two other under‐investigated directions that are essential to addressing the challenges of visualizing dense networks. While no concrete techniques were found in the survey specifically for visualizing uncertain topologies for geolocations, the typology for uncertainty in geospatial graphs by von Landesberger et al. [vLBW17] could provide useful directions. Finally, little is known about dynamic (uncertain) geospatial networks.

### 3.7 Multi‐faceted network visualization

Kehrer and Hauser [KH12] introduce the concept of multi‐faceted scientific data, where a facet is considered an aspect of the data at hand (e.g. spatio‐temporal, multi‐variate, multi‐modal, etc.). *Multi‐faceted* network visualization introduces specialized techniques for scenarios where one or more facets need to be displayed concurrently with the graph topology, incorporating together data aspects that are commonly considered distinct and non‐overlapping in other network visualization disciplines. This discipline extends general network visualization with specialized techniques for encoding multiple facets in the final representation (see Figure 1). Multi‐faceted network visualization inherits strongly from the disciplines of dynamic, multi‐variate, and group network visualization by considering these as possible facets to be represented together alongside the graph's topology.

■ **Hadlak et al**. [HSS15] present a high‐level overview of multi‐faceted network visualization (see Figure 2 for facet examples).


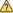
 *Motivation*: Data often comes with different aspects, whose interplay might provide useful insights during the visual exploration process. Considering the great amount of research in this field, the survey is compiled following three principles: (i) focus on the final visual result (rather than on the algorithmic means to obtain it), (ii) separate the base representation of the graph from the overlying facets and discuss their composition instead, (iii) describe a few representative examples of each composition in detail rather than providing an exhaustive list of approaches.


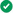
 *Contribution*: The proposed categorization separates the base representation (i.e. the primary graph facet that governs the central aspects of the composed visualization) from the composition modality (in space or time), which instead denotes how these different facets are combined together. With this categorization, the survey presents approaches for graph representation with (i) one facet, (ii) multiple facets, and (iii) multiple instances of the same facet. The survey also discusses cases of balanced representation, that is with no clearly defined base representation. When only one facet is shown along with the graph structure, several of the discussed approaches can be mapped to techniques discussed in other surveys, like dynamic network visualization for the temporal facet, group visualization for the partition facet, and multi‐variate network visualization for the attributes facet. For this reason, the survey contextualizes each facet with its relation to existing surveys, trying to provide a common terminology similar to what we do, on a larger scale, in Section 5.


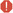
 *Open Challenges*: The authors point out that some of the categories identified by their survey appear largely under‐investigated. These include most of the temporal and balanced spatial compositions. At the time of the publication, there was no survey on geospatial network visualization published, a gap now filled by the survey from Schöttler et al. [SYPB21]. The survey also acknowledges the absence of a task taxonomy specifically oriented toward multi‐faceted graphs. Finally, hardly any visualization approaches exist for some data facets, such as provenance, uncertainty, and text.

### 3.8 Multi‐layer network visualization

*Multi‐layer* networks are defined by McGee et al. [MGM*19] as follows: starting from a graph as G=(V,E), a multi‐layer network is a triple $G_{L}=\left(V_{M},E_{M},L\right)$, where *L* is the set of layers, $V_{M}\subseteq V\times L$ is the set of vertices, and $E_{M}\subseteq V_{M}\times V_{M}$ the set of edges. This implies that each vertex can belong to one or more layers, and edges can either be inter‐ or intralayer depending on whether they connect vertices from different or the same layer, respectively. The semantics of the layers depends on the structure the multi‐layer network is designed to represent. Kivelä et al. [KAB*14] discuss in detail the theory behind multi‐layer networks and present a general framework that allows to compare and relate similar network types, such as multiplex, networks of networks, and multi‐modal networks. Schurov et al. [SM16] introduce a simple taxonomy of multi‐layer networks characterizing them according to their structural properties. Xitao et al. [XLSP17] present a short review of existing challenges in multi‐layer network visualization with some examples taken from literature. Essentially, the problem of having to visualize the dynamics, multiple attributes, and group information associated with the nodes or edges of a graph can be converted to a problem of multi‐layer network visualization, where each node or edge would be assigned to a given layer depending on the facets being depicted in its current context. Therefore, in our roadmap, all these individual facets are represented as roads converging into multi‐layer network visualization (see Figure 1).

■ **McGee et al**. [MGM*19] provide a recent review of the state‐of‐the‐art in the multi‐layer network visualization discipline.


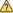
 *Motivation*: Systems dealing with data having several characteristics in common with multi‐layer networks refer to their data under different terms, such as multiplex, multi‐modal, heterogeneous, and the like. This survey aims at consolidating the work and terminology both within the field of information visualization and across application domains.


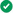
 *Contribution*: The proposed design space for multi‐layer network visualization techniques presents five dimensions: (i) task and analysis, (ii) data definition, (iii) visualization techniques, (iv) interaction approaches, and (v) empirical evaluation. The survey proposes a task taxonomy extending the previous work by Lee et al. [LPP*06] (general tasks for graphs) and Pretorius et al. [PPS14] (tasks on multi‐variate networks), citing systems exposing tasks not fitting in previous work. The data definition dimension refers to the nomenclature used in literature for techniques expressed on networks that present multi‐layer characteristics. The visualization approaches are categorized based on their awareness of the notion of a layer and are organized based on the visual encoding: from 1‐D to 3‐D node‐link representations, also discussing matrix‐based and hybrid approaches. Interaction techniques are discussed depending on whether the targets of analysis are individual network elements (nodes or edges) or entire layers or groups thereof.


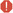
 *Open Challenges*: First, a definition of a task taxonomy specific for multi‐layer networks is missing, to motivate further research in the field. Second, the paper discusses and presents methodologies for modelling data in the form of multi‐layer networks in order to expand the potential application of its techniques to multiple domains. In terms of visualization approaches, hybrid visualization techniques are somewhat less represented in the design space and are considered an interesting direction for future work, along with novel interaction techniques and methods to encode different attributes of nodes and edges along with layer information.

## 4 Surveys Discussion

In the following section, we discuss and summarize the major open points and challenges we identified from the surveys reported in Section 3, with a specific focus on problems that crosscut and affect different disciplines. We also outline, whenever possible, their progression over time, that is if they were tackled or solved or if they still represent an open and unsolved challenge. We provide a more comprehensive overview of the major takeaways from our literature survey in Section 8.2, which also considers our task taxonomies survey and discussion.

The first surveys on graph visualization [HMM00, SS06] clearly describe the change in perspective from graph *drawing* to *visualization*, with a strong focus on visual and computational scalability, novel interaction techniques, support for hierarchies and new types of graphs. The topic of large network visualization, depicted as an open challenge in the early 2000s, became a mature discipline on its own [vLKS*11, PAKC15]: this time, with visualization regarded as a *system*, able to tackle large graphs through a series of consecutive processing stages, rather than as a monolithic layout process. Visual and computational **scalability** is as of today a major challenge in numerous disciplines [vLKS*11, AAK*14, BBDW17, SL17, SYPB21]. On the other hand, the more technical aspects of general network visualization also receive considerable attention to this day, individually focusing on layout algorithms, scalability, and aesthetic principles [GFV13, CGZ*19, CS20].

Other than using more sophisticated layout techniques, algorithms for **graph simplification** have been investigated to address the issue of visual clarity with larger graphs [HMM00, vLKS*11, GFV13, CGZ*19]. In this category, edge bundling has been proposed and thoroughly investigated as a potential solution; however, its readability and a standardized assessment of the bundling quality are still discussed as open challenges [ZXYQ13, LHT17]. Another aspect of graph simplification strategies is node clustering and aggregation [HMM00, GK10, CGZ*19]. This is an open challenge that has been exclusively discussed in the context of general network visualization; however, we see this problem extending to large, group, as well as, multi‐variate network visualization, as certain properties of the network can be leveraged in order to **improve the quality** of the clustering algorithms [AAK*14, VBW17].

Exploring the disciplines' design spaces beyond node‐link layouts is a recurring point that comes up in multiple surveys and we consider this to be a cross‐cutting challenge identified in dynamic [KKC14, BBDW17], group [SS06], multi‐variate [NMSL19], and multi‐layer network visualization [MGM*19]. These surveys outline and suggest exploring new combinations of visualization techniques as well as some of the under‐investigated categories from their respective design spaces.

**Evaluating the cognitive load** on the user during the insight‐generation process is a challenge that crosscuts general, large, and dynamic network visualization [HMM00, vLKS*11, BBDW17]. This challenge, however, has been recently investigated mostly in the context of dynamic networks [BLIC20, LPP*21], evaluating animation‐based and small multiples approaches. Alternative ways to represent a graph's temporal dynamics other than discrete time, such as **continuous or event‐based** representations, also play a major role in this intersection as performing traditional time‐slicing may hide or obscure significant behaviours and patterns that occur in the network or any of its attributes. This research direction has been formulated in the context of dynamic network visualization [BBDW17] and received increased attention recently [SAK18, AMA22]. This topic can still be explored in more detail considering how these changes can be represented in other facets of the network. Finally, it has been suggested that visualization of dynamic graphs might move towards a **confirmatory analytical modelling** stage, with the use of statistical models of network change [MMB05]. Change centrality [FPA*12] is proposed as a statistical model to perform a pairwise comparison between subsequent states of an evolving network in the discrete‐time domain for dynamic network visualization; however, more research can be conducted on this problem targeting continuous networks and intersections of different disciplines exhibiting multiple facets of the network (e.g. geospatial, group, and multi‐variate changes over time).

The problem of visualizing **multiple dynamic data dimensions** presents a bridge between general, dynamic, and multi‐variate network visualization [AAK*14, BBDW17]. These have been tackled through research on attribute‐based layouts [GFV13, NMSL19], which exploit the underlying node and/or edge variables (or characteristics) to produce a layout of the network. Another cross‐cutting challenge between these disciplines focuses on the topic of detecting and conveying **patterns in networks**. Such patterns can be captured and depicted in matrix‐based approaches by applying reordering algorithms. Reordering the rows and columns of matrix‐based representations to highlight such structures, either automatically using algorithms or leveraging interaction and human knowledge, to detect and convey these patterns is discussed as an open challenge [BBHR*16]. This same problem is also presented as a future research direction in the context of multi‐variate network visualization [AAK*14]; however, the focus here is more on node‐link representations and patterns or network motifs concerning the network's topology and its node or edge attributes.

Over time, visualization techniques specialized for **new graph types**, as well as combinations of these, were introduced, a topic that is still frequently discussed in many disciplines [SS06, vLKS*11, HSS15, BBDW17, SYPB21]. Approaches for **hypergraphs and compound dynamic graphs** are still considered as an under‐investigated open topic [vLKS*11, CGZ*19]. This concept is generalized in the context of multi‐faceted network visualization [HSS15]. In this case, the network can exhibit one or more facets simultaneously and the composition of the structural aspect along with the facets in order to effectively convey this information is considered a nontrivial problem. For multi‐faceted network visualization, specifically, a direction for future research is that some facets are still under‐investigated in literature with no dedicated approaches to representing this type of data, such as **text, uncertainty, and provenance** [HSS15].

Finally, **task taxonomies** are a major open challenge that is common among multiple surveys spanning numerous disciplines (e.g. [HSS15, MGM*19, SYPB21]). For this reason, we survey and discuss publications on this topic in Sections 6 and 7, respectively.

## 5 Consolidating Terminology

Throughout the surveys we investigated in this paper, we found several instances of similar techniques categorized over and over often using different terminology and potentially generating confusion. This poses a challenge to provide an overarching and comprehensive overview of the field. In this section, we describe our terminology consolidation with the goal of establishing a common dictionary across the different disciplines by unifying the terminology used in the surveyed literature as follows. We classify tags and keywords from each of the surveys discussed in Section 3 and group these into several categories. We look for terms referring to *similar* concepts (e.g. the terms *static flip books*, *small multiples*, refer to *juxtaposition* techniques) and the use of different wording that refers to the same concept (e.g. *network movies* to refer to *animation* techniques). We further group the categories and identify six higher‐level groups. These are: (i) facet composition, (ii) network representation, (iii) entity encoding, (iv) dimensionality, (v) Layout, and (vi) aesthetic criteria. In Table 5 we map each survey to the consolidated categories, effectively providing a *heatmap* of the most (and least) discussed concepts in recent visualization research. We discuss it in Section 8.1. The terminology consolidation and the entire list of terms is available as supplemental material and as an interactive Miro board [MIR]. Papers in our SurVis project are tagged according to our terminology consolidation and previously discussed taxonomy coverage (see our online material [PAP]). In the following, we present some relevant examples of the more popular and inconsistent categories, within their respective group. Please refer to the supplementary material for a more extensive glossary of terms and definitions [MIR].

### Network representation

*Node‐Link* diagrams visualize a network using circles for the nodes, with line segments connecting them representing the edges. In the majority of the surveys, the drawing is laid out on a 2‐D plane with straight edges. They are extremely common, widely studied, and employed, but are not ideal for all types of tasks and suffer from visual scalability issues [vLKS*11].

*Matrix* approaches visualize a graphs as a table with n×n cells, where *n* is the number of vertices. Each of the *n* ^2^ cells represents an edge between the nodes of the corresponding row and column if there is a non‐zero value present. Vertex ordering in rows/columns can be arbitrary or computed [BBHR*16]. Matrices do not suffer from visual clutter; however, they are not as effective on some tasks compared to node‐link representations (e.g. path following).

*Space‐Filling* techniques utilize the entire drawing area and implicitly encode relationships between entities by employing the principle of enclosure and proximity. These techniques are designed to visualize networks presenting a non‐overlapping hierarchy between their nodes. This type of network visualization offers benefits similar to matrix or list representations, in that, by design there is no overlap or visual clutter in the resulting visualization. However, this approach is also limited to the display of hierarchical data.

*Hybrid* approaches are defined as a mixture of techniques, combining the strengths of multiple network representations to mitigate weaknesses associated with individual approaches [vLKS*11, BBDW17]. Combinations, such as *Nodetrix* (node‐link with matrix) [HFM07], group‐in‐a‐box layouts (node‐link with treemap) [ZMC05], and matrix and tree visualization [SZEAC19] are quite popular. A number of surveys categorize these as a hybrid.

*Alternative* representations are other approaches that do not fall into the categories we outline before to visually depict networked data. Approaches in this category are usually very custom implementations of network visualization techniques typically designed with specific analytical tasks, datasets, or user groups in mind.

### Facet composition

*Superimposition* is an approach, where two or more visual representations of the network's facets are overlaid on top of each other [JE12]. The resulting visualization combines each of the facets, often needing a form of explicit encoding to distinguish between them [GAW*11] (the use of colour or other visual variables).

*Juxtaposition* is the most popular approach to depict multiple facets of networked data (e.g. temporal or multi‐variate). With juxtaposition, each of the values of a facet has a dedicated display space and the multiple visualizations are then arranged in a side‐by‐side manner, possibly ordered according to some criteria (i.e. time).

*Animation* is a dynamic representation that utilizes the physical dimension of time to convey the time‐oriented nature of the data [AMST23]. This approach is also referred to as a time‐to‐time (*dynamic*) mapping where the temporal facet of the data is mapped to simulated (*animation*) time [BBDW17]. Animation can be effective in conveying an overview of the evolution of a network and facilitating high‐level behaviour and pattern identification [BBL12].

*Timeline* is a representation that visualizes the time‐oriented nature of networked data in still images [AMST23]. Timelines utilize space to convey changes occurring to the data and are a form of time‐to‐space mapping [BBDW17] that depicts the entire evolution of a network in one or more still images [TMB02].

*Integration* is defined as placing visualizations of a network's different facets in the same view and visually linking the elements of these together [JE12]. This approach is similar to juxtaposition, with the exception that integration explicitly links the elements in each of the visualizations together relating data items from different facets (e.g. in the form of graphical lines connecting the entities).

*Nesting* is a composition where the contents of a facet's visual representation are nested inside another visualization [JE12] (also referred to as client and host visualizations). NodeTrix [HFM07] is an example of this approach, where the dense communities' topology, represented as an adjacency matrix, is nested into the node‐link visualization representing the inter‐community relationships.

*Overloading* is a form of nested visualization where a client visualization is rendered inside of a host visualization using the same spatial mapping as the host [JE12]. The client is laid over the host, as in superimposition, but there is no one‐to‐one spatial linking between the two visualizations.

*Multiple Views* refers to a composition modality describing the use of multiple (coordinated) views [Rob07]. Each window is dedicated to depicting (with its own visual representation and encoding) a specific facet of the network. Typically, this term broadly applies to multiple coordinated views, where interacting with one view would also provoke changes in other views during exploratory analysis.

### Layout

*Energy‐based* layouts refer to algorithms that minimize an energy function in order to draw the network in an aesthetically pleasing fashion. The intricacies of the layout algorithms falling into this category vary widely, most notably in their implementation of what the energy function to be minimized is.

*Heuristic* approaches for network layout involve algorithms that use a number of measurements to improve the current candidate solution (often optimizing certain aesthetic criteria) [CS20]. Such approaches query the solution space iteratively and evaluate the results of the graph layout problem optimizing for certain constraints.

*Embedding/Dimensionality Reduction* includes techniques where a high‐dimensional embedding of the network is projected into a lower‐dimensional space.

*Tabular* layouts are primarily intended for matrix network visualizations and concern the problem of finding an ordering of the rows/columns to highlight specific patterns in the data [BBHR*16] (see Section 3.1).

*Geometrical* approaches refer to techniques whose network representations satisfy specific geometrical constraints. Such techniques are tightly coupled with the structure and properties of the networked data and elicit visual representations of the graph emphasizing a certain topology (i.e. planar graphs, trees, or hierarchies [SSK14]).

*Special‐Purpose* visualization techniques have been applied to different types of networks to support specific tasks, e.g. represent time in a dynamic graph [SL17, BBDW17], represent the value of an attribute of the node [NMSL19], or the geospatial position of a node [SYPB21].

## 6 Task Taxonomies

**Figure 9:**
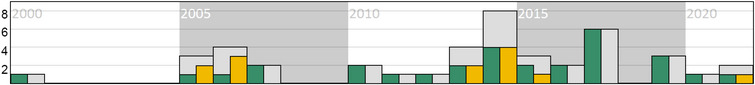
Distribution of surveys (green) in relation to task taxonomies (yellow) over the years on the topic of network visualization. The surrounding bars (gray) accumulate the total count of both task taxonomies and surveys. See [PAP] for an interactive version.

In this section, we propose an in‐depth description of the task taxonomies we found during our literature search. For each one of them, we highlight its motivation, outline the taxonomy structure, and briefly discuss its evaluation and overall contribution to the field using the same structure as we did with surveys in Section 3. Figure 9 depicts the distribution of taxonomy papers over time in relation to the number of surveys published.

### 6.1 Higher level visualization taxonomies

Several of the task taxonomies relevant to this meta‐survey are based on general task taxonomies for visualization. While not strictly in our survey scope, we shortly summarize their concepts to ease the reading of the following sections.

■ **Amar et al**. [AES05] presents a set of low‐level analysis tasks that describe users' activities when faced with information visualization tools to better understand data. These tasks are abstract and can be considered agnostic of the visualization techniques and the data characteristics. The paper presents ten component tasks: (i) retrieve value, (ii) filter, (iii) compute derived value, (iv) find extremum, (v) sort, (vi) determine range, (vii) characterize distribution, (viii) find anomalies, (ix) cluster, and (x) correlate.

■ **Adrienko and Adrienko** [AA06] introduce the concepts of *referrer*, to describe data that indicates context (the value of the referrer is called a *reference*), and *attribute* for data representing measurements (the value of the attributes is called a *characteristic*). With this definition, a dataset consists of a set of references, a set of characteristics, and a data function defining the relationship between them. The task taxonomy proposes to divide the tasks into elementary and synoptic tasks. Elementary tasks are centred around individual elements and include (i) lookup, (ii) comparison, and (iii) relation‐seeking operations. Synoptic tasks involve (sub‐)sets of references and can be further subdivided into descriptive (lookup, comparison, and relation seeking) and connectional (support understanding relationships between the data characteristics).

■ **Brehmer and Munzner** [BM13] present a more abstract visualization task taxonomy, centred around three questions: (i) *why* is the task performed, as a combination of action and targets (e.g. consume, produce, search, and query), (ii) *how* is the task performed (e.g. manipulate, select, introduce), and (iii) *what* are the task input(s) and output(s). This structure supports analysts in performing complex visualization tasks as a sequence of intermediate low‐level operations and can model dependencies between them by using the output of a prior task as the input for subsequent ones.

■ **Valiati et al**. [VPF06] propose a taxonomy of high‐level visualization tasks to support exploratory or statistical analysis in multi‐dimensional (multi‐variate, in this context) visualizations. It is based on previous work in this domain [WL90, SBM92] as well as the results obtained by an internal user study. The proposed taxonomy classifies tasks into seven high‐level categories: (i) identify, (ii) determine, (iii) visualize, (iv) compare, (v) infer, (vi) configure, and (vii) locate. The first five tasks express analytical goals, that is analysing some statistical properties of the dataset, with the other two being intermediate‐level tasks that support them.

■ **Roth** [Rot13] presents an empirically derived task taxonomy for geovisualization and interactive cartography. The paper proposes tasks that can be expressed through the following interaction primitives [Nor88]: (i) the goal (procure, predict, prescribe), (ii) the objective (identify, compare, rank, associate, delineate), and (iii) the operator (function to support the objective). A task can be considered a combination of *goal*+*objective*+*operator* to be applied on an operand, which can be individual geographic components or characteristics of geographic phenomena in space or over time. Operators can be *enabling* (e.g. import, save, edit), such that they prepare or clean up for a *work* operator (e.g. sequence, arrange, search) that actually accomplishes the objective.

### 6.2 General network visualization

■ **Lee et al**. [LPP*06] present a task taxonomy for graph visualization to support designers and evaluators in creating and comparing techniques on a standardized set of tasks.


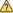
 *Motivation*: At the time this taxonomy was published, only a limited number of techniques had been tested through user studies. To motivate and support comparative evaluations, the paper proposes a task taxonomy with the aim of allowing researchers to draw generalizable conclusions from their experiments.


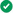
 *Contribution*: The proposed tasks, commonly encountered when analysing graph data, can be classified as compound tasks made up of the low‐level tasks proposed by Amar et al. [AES05], which have been expanded to include graph‐specific objects. The proposed taxonomy breaks down the tasks into four main categories: (i) topology‐based, (ii) attribute‐based, (iii) browsing, and (iv) overview. The topology‐based task category includes low‐level graph tasks such as adjacency (finding the set of nodes adjacent to a node), accessibility (find the set of nodes accessible from a node), connectivity (find the shortest path between two nodes), and common connection (given nodes, find the set of nodes that are connected to all of them). Attribute‐based tasks can be performed on either the nodes or links of a network, such as finding nodes having a specific attribute value, finding nodes connected only by a certain type of link, or finding nodes connected by links having low/high value. Browsing tasks are more of a compound task as they involve multiple lower‐level tasks, such as, following paths or scanning a set of nodes and tracing their paths to find adjacent nodes and structures. Finally, the overview task category is described as a compound exploratory task with the goal of estimating the size of a network or different entities (such as clusters, groups, or connected components).

Despite not presenting an evaluation, this general taxonomy withstood the test of time, inspiring several other taxonomies [BDA*17, SSK14] and providing a solid base for controlled experiments on graph visualization systems (see, e.g. the eye‐tracking experiment by Pohl et al. [PSD09]).


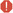
 *Open Challenges*: The paper recommends the definition of benchmark datasets would be helpful in supporting evaluations and generalizing their results. Moreover, it describes the need for further classification of network visualization approaches, based on which properties of the graph they visualize (i.e. directed graphs, dense or sparse graphs). This was actually achieved in the following years, as research in network visualization branched into different disciplines, each focused on a specific type of networked data as we describe in this survey.

#### Tree visualization

■ **Pandey et al**. [PSS*21] propose a task taxonomy for tree visualization.


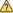
 *Motivation*: The absence of a task taxonomy dedicated to the visualization of trees presents a significant challenge when developing and creating novel visualization techniques for this data type, as there are no tasks to guide the design. Furthermore, evaluating the proposed approaches in terms of the effectiveness of the visual encodings and interaction techniques also becomes an obstacle due to the lack of a well‐defined task taxonomy.


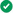
 *Contribution*: The task taxonomy is presented as an extension of the multi‐level task typology by Brehmer and Munzner [BM13] (see Section 6.1) with new elements in the *why* category. The *why* is extended with *analyse*, *search*, and *query* actions alongside a hierarchical classification of targets. The highest level of this task target hierarchy can refer to either the tree's topology or attributes. Mid‐level specific targets identify which tree structure is the target, ranging from larger parts of the tree down to specific elements (i.e. tree, subtree, path, nodes). Finally, for each specific target, distinct attributes are specified. The final result is a flexible taxonomy, that easily allows for classifying tasks on trees, for example “Find the height of the tree” would be classified as *topology* target, *tree* as a specific target, *height* as the attribute. The paper also describes the creation of curated datasets for researchers and practitioners and use case scenarios, including the design of an evaluation study and the exploration of visual encodings that are suitable for the tasks in the taxonomy.


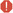
 *Open Challenges*: The paper describes research opportunities for tree visualization systems, highlighting the lack of crowd‐sourced studies, and discussing underrepresented tasks and diagrams in the proposed design space. Future work in task research for trees includes, first and foremost, addressing the inconsistencies in task phrasing, similar to what drove us in consolidating the terminology in our work. Second, introducing task abstraction guidelines would enable the comparison of tasks from a wide range of papers through a standardized vocabulary. Finally, artificial intelligence might help practitioners to validate task abstraction designs.

### 6.3 Group structures visualization

■ **Saket et al**. [SSK14] introduce a task taxonomy for the visualization of group structures in networks.


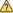
 *Motivation*: The paper addresses the lack of a taxonomy for approaches that specifically tackle the visualization of groupings and hierarchies within networks.


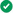
 *Contribution*: The paper presents a task taxonomy for graphs with additional grouping information. Previous work by Amar et al. [AES05], Lee et al. [LPP*06], and Brehmer and Munzner [BM13] provide the basic framework and terminology for this taxonomy. The paper lists 29 tasks, obtained from both studies on user interaction with visualization and from interviews with experts in the field. These are divided into four categories according to the information required to solve them: (i) group‐only tasks require only group information (“Which group has the maximum number of neighbouring groups?”); (ii) group‐node require group and node information (“Count the number of nodes in a group”); (iii) group‐link require group and edge information (“Count the number of links in a group”); and (iv) group‐network require group, node, and edge information (“Find a group that has the node with the lowest/highest degree”). Graphs are assumed as simple and undirected, but most of the presented tasks can be easily generalized to both weighted and directed graphs. Low‐level tasks can also be combined to describe more complex, higher‐level tasks. The taxonomy is not evaluated; however, the paper presents some considerations on how these tasks can be used in evaluation studies.


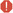
 *Open Challenges*: In terms of future work the paper discusses the possibility of extending the proposed task taxonomy and further specializing it for more specific data types. A specific example is to expand it to consider more complex grouping structures, such as overlapping groups or multi‐level clustering.

### 6.4 Dynamic network visualization

■ **Ahn et al**. [APS14] introduce a task taxonomy for the analysis of network evolution.


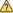
 *Motivation*: The paper proposes a task taxonomy aimed at supporting research in the visualization of temporal networks. This taxonomy addresses the lack of suitable analysis methods to guide the development and design of visualization approaches for depicting the network's evolution.


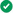
 *Contribution*: The taxonomy proposes a comprehensive framework of tasks to visualize dynamic networks. To build the taxonomy, the authors collected, reviewed, and categorized tasks from existing temporal visualization systems. The taxonomy develops over three dimensions: first, the *Entities*, that define the object of interest, ranging from individual nodes and edges to groups, to the entire network. Second, the *Temporal Features*, that define how to observe, identify, or compare the entities' status over time and can be related to individual events (e.g. single occurrences, replacements, (dis‐)appearances) or aggregated events (e.g. the *shape* or *rate* of changes). Third, the *Properties*, that are associated with entities, can be either structural properties (attributes from the topology of the network) or domain‐specific properties (attributes independent of the network structure). The taxonomy was validated by means of an expert evaluation with 12 participants.


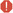
 *Open Challenges*: The design process behind this taxonomy unveiled several research opportunities and lessons learned. When mapping the reviewed visualization systems to the task design space, it shows that while domain properties prevail (i.e. almost all proposed tasks incorporated domain properties), the *rate of changes* is scarcely explored. Individual events are preferred over aggregated events, except for simpler ones, like growth and contraction. Similarly, compound tasks were not as explored as low‐level tasks. Finally, few studies provide means to control the visual analysis granularity, for example node/link, group, and network level.

■ **Bach et al**. [BPF14] propose a task taxonomy for dynamic networks in the context of their work GraphDiaries.


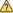
 *Motivation*: The authors discuss related task taxonomies, such as the work by Amar et al. [AES05], Lee et al. [LPP*06], Adrienko and Adrienko [AA06], and Ahn et al. [APS14]; however, for their problem and context these were considered too complex and focused on *analysis* of networks. Therefore, motivating the need for a simple, systematic taxonomy to understand changes happening and support temporal navigation in dynamic network visualization.


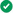
 *Contribution*: The proposed taxonomy is inspired by the work of Lee et al. [LPP*06] combined with a framework for geo‐temporal tasks by Peuquet [Peu94]. Peuquet mentions three dimensions: *when*, *what*, and *where*. In this taxonomy, the tasks are centred around finding the value of the third dimension (e.g. “when”) at the intersection of the other two dimensions (e.g. “what” and “where”). As Peuquet's taxonomy is developed with fixed geographical locations in mind, to adapt it for dynamic networks (i.e. where the position of the nodes can change over time), the *where* and *what* dimensions have been slightly redefined. Specifically, the geographical *where* definition is replaced by nodes, edges, and higher‐level topological structures as described by Lee et al. [LPP*06]. The *what* dimension has been redefined to capture the type of change (e.g. appear/disappear) and the behaviour of network elements. With this definition of low‐level tasks, it is straightforward to combine them and form compound and higher‐level tasks.


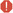
 *Open Challenges*: With regards to the proposed task taxonomy the authors do not elaborate on any new directions for future research. However, they state that this task taxonomy can guide the development and design of interfaces for temporal exploration and navigation in dynamic networks. Future work is centred around the extension of their approach, *GraphDiaries*, to consider evolving multi‐variate and hierarchical graphs and effectively visualizing the evolution of nodes and edge attributes.

■ **Kerracher et al**. [KKC15] present a taxonomy for dynamic (*temporal* in the paper) graph visualization.


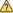
 *Motivation*: It is not always possible to know the users' tasks a priori; therefore, designers and developers should provide generic approaches supporting a wider range of tasks, which is the main motivation behind the proposed taxonomy. The authors consider the task framework from Adrienko and Adrienko [AA06] in the context of temporal graph visualization, as it offers a flexible structure and a wide range of tasks.


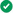
 *Contribution*: The authors extend both the data model and the task framework from previous work [AA06] to account for graph data. The data model is augmented with *graph* as a new referrer (see Section 6.1) and *nodes* as its members. A new type of relationship, *linking*, is introduced to encode the edges between the nodes in the graph. *Linking* relationships can change over time in terms of their existence and domain properties. This requires two new concepts to capture the variations in the graph structure: structural behaviour and structural pattern. They represent graph objects (paths, subgraphs, etc.) and notable graph structures (clusters, cliques, etc.), respectively. The task framework is extended with *attribute‐based* and *structural* tasks, to support investigating these new structural behaviours. Each category follows the elementary and synoptic task distinction of the original framework [AA06]. The authors further categorize temporal graph data, resulting in a two‐dimensional taxonomy of synoptic tasks, with the first dimension being the time duration (instant or interval) and the second being the scope of the graph structure (single element or sets of elements). Kerracher et al. [KKC15] also introduce connection discovery tasks, aimed at finding indications of relations between parts of a single or multiple phenomena. These tasks support investigating what effect the graph structure has on its attribute values and how some structural patterns affect others at different points in time. Both the methodology and the coverage of the proposed task taxonomy were evaluated by means of comparing them with similar existing work, such as the taxonomies by Bach et al. [BPF14] and Ahn et al. [APS14].


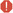
 *Open Challenges*: For future work the authors propose conducting user studies assessing the most important tasks in the proposed taxonomy, as well as, categorizing visualization techniques according to their task support. The latter has already been investigated in follow‐up work by the authors [KKCG15].

### 6.5 Multi‐variate network visualization

■ **Pretorius et al**. [PPS14] suggest a set of tasks for multi‐variate network analysis.


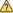
 *Motivation*: Constructing a frame of reference for multi‐variate network visualization as well as describing user tasks in this domain presents the motivation behind the proposed task taxonomy. The intent of this work is to model tasks with the goal of gaining insights and conducting multi‐variate network analysis, not solely focusing on the visual representation of such networks.


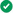
 *Contribution*: The authors describe tasks as analytic processes carried out by users and applied to a specific entity's property. The presented taxonomy is built upon the work by Lee et al. [LPP*06] and heavily based on the work by Valiati et al. [VPF06]. The proposed taxonomy mirrors the structure by Lee et al. [LPP*06], categorizing tasks in topology‐, attribute‐based, browsing, and overview. Small changes of terminology are applied, such as topology‐based tasks becoming *structure‐based* and overview tasks becoming *estimation*. The proposed tasks can be considered as compositions of lower‐level tasks from the taxonomy of Valiati et al. [VPF06] (see Section 6.1). Examples of tasks from this taxonomy include finding a node (or cluster) with specific attribute values or characterizing the set of nodes as belonging to different groups based on link attributes. This task taxonomy, therefore, comes up as a synthesis of existing frameworks. A limitation of this approach is that it has not been designed with the user's prior domain knowledge of the target attributes in mind.


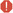
 *Open Challenges*: Future work is mostly concerned with the implication of prior knowledge (i.e. how existing knowledge on node, edge, or group properties affects the execution of tasks) on the resulting task taxonomy. Including more semantic and situational analysis of multi‐variate networks can potentially change the way that the tasks are conducted and how the results are interpreted. Finally, the authors propose to evaluate this taxonomy and determine its suitability for multi‐variate network analysis.

## 7 Taxonomies Discussion

In Section 6 we survey and describe each of the existing task taxonomies on network visualization in the scope of our meta‐survey according to our inclusion rules (see Section 2.2). In this section, we examine this body of research focusing on a more high‐level perspective of the current literature. Specifically, we aim at mapping and outlining the most and least supported disciplines by existing task taxonomies, providing potential directions on what has yet to be done in this direction. To do so, we introduce the notion of taxonomy coverage in Section 7.1 and use it to describe saturated network visualization disciplines as well as to outline how to leverage existing research to build a taxonomy for the disciplines that are currently lacking one. We conclude the section by compiling a more granular classification of tasks across all taxonomies, clearly showing the most and least targeted aspects from the available tasks across different disciplines (Section 7.2).

### 7.1 Taxonomy coverage

In our literature search, we found surveys that explicitly acknowledge the lack of a taxonomy for their discipline and consider this an open challenge, whereas others argue how existing taxonomies can be adapted and extended to fit the analytical tasks in their domain. We investigate this matter to precisely identify the gaps in related literature and, therefore, introduce the concepts of *specialized* and *generalized* taxonomy support, which we use in the following to categorize, as shown in Table 3, the papers presented in Section 6.

**Table 3:** Task taxonomies are classified according to their related network visualization discipline and type of support provided.

	Discipline	General	Large	Group	Dynamic	Multi‐variate	Geospatial	Multi‐faceted	Multi‐layer
**Coverage**	**Specialized**	[LPP*06]	–	[SSK14]	[BPF14], [APS14], [KKC15]	[PPS14]	–	–	–
	**Generalized**	[AES05], [BM13], [PSS*21]	[LPP*06]	[LPP*06], [APS14], [SSK14]	[LPP*06], [AA06]	[AES05], [LPP*06], [AA06], [VPF06], [BM13]	[LPP*06], [AA06], [Rot13], [BPF14]	[LPP*06], [AES05]	[AES05], [LPP*06], [BM13], [PPS14], [APS14]

**Specialized** taxonomy support indicates that they are designed to include tasks that target specific data properties of their corresponding network visualization discipline. In this case, we conclude that a taxonomy provides “specialized” support for a specific discipline. Such taxonomies might not be as relevant for applications outside of their intended discipline (e.g. specific dynamic network tasks might not be applicable to general network visualization). In this category the task taxonomies are well‐established and highly cited publications that have been or that can readily be used in formal evaluation settings or design studies. The majority of taxonomies we discuss in Section 6 are specialized to support their own discipline: for example, the works by Ahn et al. [APS14] and Kerracher et al. [KKC15] (see Section 6.4) offer specialized support for dynamic network visualization. Similarly, the taxonomy by Pretorius et al. [PPS14] offers specialized support for multi‐variate networks.

**Generalized** taxonomy support includes more abstract visualization tasks, intended to cover a broader range of data types and characteristics or ones whose tasks abstract or overlap with other disciplines. Therefore, a taxonomy that offers generalized support for a discipline might not provide a comprehensive and fully expressive set of tasks for that specific data type, but can be used as a basis for deriving new tasks or adapting existing ones. We mapped the generalized taxonomy support to each discipline based on two criteria: (i) if a taxonomy, offering specialized support for a discipline, extends or takes inspiration from another, the latter provides generalized support for the same discipline; (ii) if a survey references a taxonomy as a potential source of tasks for the discipline (as typically happens with higher‐level taxonomies, see Section 6.1), this reference is considered to offer generalized support.

Within this context, the most widely used taxonomies are the ones by Amar et al. [AES05] and Lee et al. [LPP*06]. These were often used as building blocks and inspiration for the development of other task taxonomies. Therefore, they offer generalized support for a wide range of disciplines, including general, multi‐faceted, group, dynamic, multi‐variate, geospatial, and multi‐layer network visualization. Adrienko and Adrienko [AA06] propose a generalized taxonomy for the disciplines of dynamic, multi‐variate, and geospatial network visualization. The work of Ahn et al. [APS14] is referenced by Hadlak et al. [HSS15] as a generalized taxonomy supporting multi‐faceted network visualization. Similarly, McGee et al. [MGM*19] reference the work by Ahn et al. [APS14] and Pretorius et al. [PPS14] in the scope of multi‐layer network visualization. Valiati et al.'s [VPF06] taxonomy is used in multi‐variate network visualization [NMSL19] as a starting point for multi‐variate network analysis tasks. Brehmer and Munzner [BM13] complement existing task taxonomies and their work has been an inspiration for developing taxonomies in the disciplines of general, multi‐variate, and multi‐layer network visualization. Finally, the work proposed by Roth [Rot13] for geovisualization and interactive cartography can be used as a basis for developing a well‐established task taxonomy for geospatial networks [SYPB21]. These relationships match and contribute to the roadmap we presented in Section 2.2.

### 7.2 Task classification

In this section we provide a classification of individual tasks from the taxonomies and structure these into three categories identified from our literature search. For a more detailed view of how all the tasks can be related and their overlaps, we refer to the miro board available as supplemental material [MIR]. From our set of surveyed task taxonomies (see Section 6) we could identify three main types of tasks that are common amongst all of the taxonomies and used in designing evaluations and studies. We discuss these categories in more detail in the following, excluding the higher‐level visualization taxonomies introduced in Section 6.1 as they are not in our meta‐survey scope (see Section 2.2).

**Topology** tasks are concerned with operations that can be performed on individual entities of a graph, including nodes (e.g. “Find adjacent nodes”), links (e.g. “Find the shortest path between two nodes”, “Follow a given path”), sub‐networks, such as groups, partitions, clusters, cliques (e.g. “Identify clusters”, “Are the given two groups neighbours”), and the entire network (e.g. “Estimate the size of the network”). Most tasks in this category involve adjacency, accessibility, identifying common connections, and connectivity [LPP*06, PPS14]. The tasks described in this category are low‐level tasks conducted on specific entities in the network.

**Analytic activity** tasks can be considered as higher‐level tasks and subdivided into analytical tasks, operational tasks, and cognitive tasks [VPF06, PPS14]. Analytical tasks describe goals that involve visually exploring or analysing a dataset using statistics and metrics (e.g. “Find/discover/estimate properties, categories, or characteristics”, “Calculate and derive statistical properties and scores”, “Compare and analyse data, items, and values”). Operational tasks are intermediate tasks that support the analysis through operations such as visualizing a dataset or re‐configuring and tuning parameters or visualization properties (e.g. “Configure parameters of the classification”, “Reorder rows and columns of a matrix”, “Select graphical primitives”, “Re‐encode the network”), and operations that can be performed on the visual representation (e.g. “Manipulation”, “Search/Query”, “Produce/Introduce”). Cognitive tasks involve extracting knowledge and insights from the network itself in terms of identifying patterns, describing changes, and inferring causal relationships (e.g. “Describe the change in behaviour”, “Analyse dependencies between changes”, “Find/Describe trends”, “Validate hypotheses”, and “Infer cause and effect relationships”).

**Facet** tasks describe operations that can be performed on the different facets of a network (as discussed by Hadlak et al. [HSS15]), such as, the temporal (e.g. “Observe if an entity appears or disappears”, “Find if and when an edge direction changes”), geospatial, multi‐variate (e.g. “Identify nodes/links with a specific attribute value”, “Which node is connected by a link having largest/smallest value”), and group facets (e.g. “Given two groups find the set of common group neighbours”). In the taxonomies, we surveyed there were no mentions of tasks relating to the geospatial facet and in the previous section, this gap was also discussed (see Section 7.1).

In Table 4 we map task taxonomies to the aforementioned categories. All task taxonomies extensively cover topology‐centred tasks on nodes and links, whereas the tasks that can be performed on sub‐networks (e.g. clusters, groups) and on the entire network did not receive the same widespread support. The taxonomy by Pretorius et al. [PPS14] is the only one to introduce operational tasks. We conjecture this is due to the specific representation of a task as an analytical *process* introduced in the paper: as such, it is reasonable to introduce a set of operations that do not achieve a result in the analysis process, but rather support other tasks. Conversely, all taxonomies present tasks belonging to the analytic category, confirming this category to be the most prominent and considered the most important across the board. Cognitive tasks are high‐level tasks that are only partially covered by the literature we survey. Concerning facet‐related tasks, several taxonomies do cover the multi‐variate category, as it includes general tasks involving graph attributes. Temporal facets are also more populated, compared to the group and geospatial ones, as the temporal facet (i.e. dynamic networks) received substantially more interest than the geospatial or groups facet. The complete lack of a task taxonomy for geospatial networks was identified as an open challenge by the recent survey by Schöttler et al. [SYPB21]. Group facet tasks, included in the taxonomies by Saket et al. [SSK14] and Kerracher [KKC15] et al., focus on the relationships and identification of groups and their attributes at different granularities (i.e. single node, clique/cluster).

**Table 4:** Our proposed task classification. Each dot (cell) represents a taxonomy (column) presenting tasks belonging to a category (row).

Tasks		Taxonomies						
		[LPP*06]	[APS14]	[PPS14]	[SSK14]	[BPF14]	[KKC15]	[PSS*21]
**Topology**	**Nodes**	•	•	•	•	•	•	•
	**Links**	•	•	•	•	•	•	•
	**Sub‐networks**	•	•		•	•	•	•
	**Network**		•	•	•		•	•
**Analytic activity**	**Operational**			•				
	**Analytical**	•	•	•	•	•	•	•
	**Cognitive**		•	•		•	•	
**Facet**	**Time**		•			•	•	
	**Space**							
	**Multi‐variate**	•	•	•	•			•
	**Groups**				•		•	

Finally, most task taxonomies consider and discuss compound tasks as well, defined as combinations of multiple low‐level tasks forming more complex paths of interaction and analysis. In the categorization proposed in this section, we do not consider any of the categories in Table 4 to be mutually exclusive. Therefore, in the context of creating complex, high‐level, and cross‐discipline tasks, these categories can be combined with each other (e.g. combining topological tasks on nodes with the multi‐variate facet for an analytical activity to cluster similar nodes together).

## 8 Discussion

In this section, we provide a high‐level discussion about the consolidation, report and discuss the most prominent takeaways, and we review the limitations of our survey.

### 8.1 Terminology consolidation

In Section 5 we discuss how we produce the heatmap in Table 5, which provides insights about the research landscape in the field of network visualization. This provides a high‐level indicator of the most researched concepts in literature according to the surveys included in this paper. If a survey is present in the table, then it includes papers that introduce terms we use in our consolidation. In turn, grey cells *do not* represent a lack of research in that direction, but rather indicate that in our literature review, there are no surveys whose terms fall into that category.

**Table 5:** Our proposed terminology consolidation. In each cell, we group references to surveys that present concepts of the consolidated terminology (rows) applied each discipline (columns). Cells colours encode the amount of papers for each discipline and term (from yellow to red). Gray denotes empty cells, which means that none of the surveys listed in Table 2 discusses that combination of terminology/discipline.

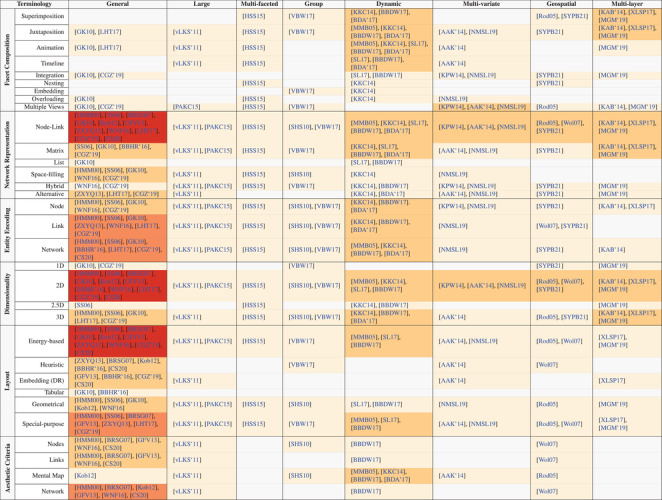

A quick, visual analysis of the table shows that the discipline of general network visualization, which is also the most dated, comprises the majority of the available research. Within this discipline, 2‐D node‐link representations, drawn using an energy‐based algorithm, appear to be the most discussed topics. Conversely, few papers discuss facet composition, which is expected as general network visualization does not encompass the representation of further dimensions.

Dynamic network visualization is the second discipline, in terms of number of surveys. The majority of research focuses on the temporal facet composition, specifically on techniques ranging from juxtaposition to animation. Other methods have been explored [KKC14], with the exception of “multiple views” that has not been investigated, in this context, as a composition method for the time facet. Concerning the network representation, there is more balance between node‐link and matrix representations. In terms of layout, energy‐based is still the most popular, possibly due to the fact that many techniques extend visualization approaches for static graphs. We also note that the *mental map* category is also the most researched aesthetic criterion in this field.

Another observation that we can make, based on the surveyed research, is that network visualization disciplines tend to experiment differently with facet composition methods. This can be seen by the non‐uniform distribution of grey cells within the facet composition category across different disciplines. It is also clear that very little research is done on aesthetic criteria in the multi‐faceted, multi‐variate, and multi‐layer disciplines. We argue this to be due to the fact that research on disciplines other than general network visualization is more application‐oriented, and therefore, can be harder to extract concepts that can be generalized in terms of readability and human perception. Moreover, it is also possible that as the other disciplines stem from general network visualization, its concepts about aesthetics are still valid also in other disciplines.

### 8.2 Takeaways

With our comprehensive analysis of the field of network visualization, from surveys to task taxonomies, we could identify some takeaways that represent promising opportunities for further research. For a comprehensive list we refer to our online material [MIR].

#### Potential of matrices

Several surveys point out how tabular techniques present an interesting yet underdeveloped potential for network visualization. As matrix‐based representations of networks present several advantages compared to the more popular node‐link approaches, they have been highlighted as promising future research directions for the visualization of dynamic and multi‐variate networks [KKC14, BBDW17, NMSL19]. The use of this representation has not been experimented with in all network visualization disciplines (see Table 5), leaving several future work opportunities, especially considering the use of existing reordering techniques.

#### Hybrid and alternative approaches for complex data

The size, heterogeneity, and dimensionality of networked data are on the rise. However, this complexity also presents opportunities for novel contributions, specifically, hybrid and alternate representations for such complex networked data should be investigated, to overcome the limitations of standard approaches (e.g. scalability). Research on alternate visualization methods also renewed the drive and motivation for recent developments in immersive network analytics [FP19]. Innovative metaphors to visualize networked data have been pointed out by selected surveys as an interesting direction for future research [SHS10, BBDW17, MGM*19]. Furthermore, the approaches that we have grouped together as “Alternative” in our consolidation table (see Table 5) are not an exhaustive list, meaning that the design space of hybrid and alternative network representations has not been explored to its full potential.

#### Task taxonomies and evaluations

Our meta‐survey shows that while extensive research has been done on task taxonomies, there are still several disciplines that lack a set of tasks specifically designed for them [HSS15, MGM*19, SYPB21] (see Section 7.1 and Table 3). Filling these gaps would facilitate the formal evaluation and comparison of network visualization techniques. Specifically, as network visualization systems become more complex, several surveys push for surpassing the traditional performance metrics (time and accuracy) and instead focus on the evaluation of cognitive aspects, perception issues, and user engagement [HMM00, Rod05, GK10, ZXYQ13, LHT17, BBDW17].

#### Interaction techniques

Approaches to facilitate navigation, exploration, and interaction with the network and its elements are highlighted by a number of reports as a promising direction for future work [vLKS*11, PAKC15, BBDW17, MGM*19]. They are a central point of discussion that spans multiple disciplines ranging from general [BBHR*16], to large [vLKS*11, PAKC15], to dynamic [BBDW17], to multi‐layer [MGM*19] network visualization. Specifically, techniques that enable interactive graph simplification and layouts have been mentioned multiple times in our surveyed literature [vLKS*11, LHT17]. Moreover, there is growing interest in systems that implement novel interaction techniques for network exploration on large [LAN20] and small tactile displays (e.g. [DLDBI10, HL07]), including real‐time collaboration during the network analysis process [CGZ*19]. Additionally, human‐assisted approaches to combine the knowledge of domain experts with automated analysis and visualization of networked data are considered to be an increasingly important yet under‐investigated research direction [BBHR*16, VBW17]. We believe this still to be an open and unresolved issue with great potential for future work, as there is no well‐defined taxonomy or survey on interactions for network visualization with few exceptions, such as the work by Wybrow et al. [WEF*14] on interactions on multi‐variate networks. These play a very important role in collaborative network analysis, a topic that has been recently gaining traction [LAN20].

#### Restructuring and unifying

Our meta‐survey shows the significant breadth of concepts, terminology, and research related to network visualization. Several concepts span multiple disciplines and we also experienced how small changes (or overlaps) of terminology might confuse and mislead. With this large body of knowledge, we believe we have reached a point in network visualization research, where we should consider taking a step back and reassessing the problem from a broader perspective. As this field begins to face the challenges offered by machine learning and immersive analytics, “de‐fragmenting” and unifying the existing research would increase the awareness and knowledge of the available technologies and theory. Our roadmap is intended as a first step in this direction.

#### Uncertainty of networks

Uncertainty is an inherent property of the data, due to errors, noise, or unreliable sources. It propagates throughout the analytical process, ultimately affecting the decision‐making. Addressing and visualizing uncertainty in network visualization is a topic of major interest among and across several disciplines and is a research field rapidly gaining traction [vLKS*11, HSS15, SYPB21]. Specifically, visualizing uncertainty associated with the network topology [vLKS*11, SYPB21], the attributes of its entities [vLKS*11], the temporal information [SYPB21], dynamically changing groups [VBAW15] is still a challenging topic and opportunity for future research. In the context of group network visualization [VBW17], the effective depiction of fuzzy and overlapping group structures is outlined as a challenge and is still not exhaustively explored in literature [VRW13].

#### Hypergraph visualization

Several surveys identify that limited research has been done on the topic of hypergraph visualization [SS06, vLKS*11, MGM*19]. This type of network structure, where an edge can connect more than two vertices, has been used in biology and image retrieval using machine learning [FFKS21]. Hypergraphs better capture group structures and many‐to‐many relationships and preserve the ability to encode other attributes (i.e. directed or weighted edges). Interest in this topic is on the rise, with the foundations of a survey in this discipline already laid out by Fischer et al. [FFKS21].

### 8.3 Limitations

Although we provide a detailed overview of the available surveys on network visualization, there are also inherent limitations of this work that must be considered. The number of surveys is relatively small, especially when compared to McNabb and Laramee meta‐survey [ML17]. However, that work surveyed the much broader field of Information Visualization, while we have a specific focus on networks only. In our meta‐survey, we excluded most algorithmic and theoretical contributions which would require a survey paper on their own. Graph theory is a huge topic and our focus on visualization‐specific aspects allowed us to provide a descriptive overview of the aspects of the field that we believe to be of the most interest to the intended audience of this journal.

Our classification is based on network visualization disciplines and their definitions are based on the respective data type. This proved to be a reasonable approach, as the majority of papers could be assigned unambiguously to each category. However, this resulted in the general network discipline being the largest one (in terms of assigned papers), and made some classifications problematic. A notable example is the paper by Schulz et al. [SHS10] about implicit hierarchy visualization. Hierarchies can be represented as trees and, therefore, tree visualization techniques can be applied in this context as well. However, we believe group visualization is a more appropriate category for this survey as the semantic (i.e. inclusion) of the edges in hierarchies is not as general as the one intended for trees. Future extensions of this work might address this ambiguity, and other potential biases introduced during the paper selection and classification, by categorizing papers differently. We envision a further analysis of research in this field from a technique perspective (rather than from a survey basis), where each is discussed and classified based on their extensions and applicability to different disciplines, possibly building upon our work in Table 5.

## 9 Summary and Conclusion

In this work, we presented our meta‐survey of the field of network visualization with the goal of providing a roadmap detailing and structuring the different research directions that network visualization has branched into. We examined both state‐of‐the‐art reports and task taxonomies related to the field in order to provide a broad overview, highlighting more and less saturated research directions, and outlining disciplines of network visualization that do not have well‐established task taxonomies to guide evaluations and comparisons of techniques. Furthermore, we discussed the inconsistencies of the terminology used throughout the surveys and consolidated these with the intention of supporting a common dictionary for the field of network visualization. Finally, we proposed a summary of the ongoing challenges and directions for promising research opportunities. Based on the results of our meta‐survey, we found that the field of network visualization is still rapidly expanding and branching into different domains. We can state that there is still much to be done in terms of novel visualization metaphors, evaluation methodologies, interaction techniques, and collaborative analysis. The answer to the overarching question of this work “*Are we there yet?*” being “*For the last time no… and stop asking*.”.
